# Hammerhead ribozymes directed against mRNA of an essential gene inhibit *Escherichia coli* growth and enhance tetracycline efficacy

**DOI:** 10.3389/fmicb.2025.1663476

**Published:** 2025-10-03

**Authors:** Joanna Miszkiewicz-Golec, Ksenia Maximowa, Maciej Łukaszewicz, Dariusz Bartosik, Edward Darżynkiewicz, Joanna Trylska

**Affiliations:** ^1^Centre of New Technologies, University of Warsaw, Warsaw, Poland; ^2^Division of Biophysics, Faculty of Physics, Institute of Experimental Physics, University of Warsaw, Warsaw, Poland; ^3^Department of Bacterial Genetics, Faculty of Biology, Institute of Microbiology, University of Warsaw, Warsaw, Poland

**Keywords:** hammerhead ribozyme, catalytic RNA, RNA-based antimicrobials, bacterial mRNA targeting, translation inhibition, acyl carrier protein, *Escherichia coli*

## Abstract

Aiming to find novel ways to inhibit bacterial growth, we tested hammerhead ribozymes targeting the mRNA*_acpP_* transcript, which encodes the essential acyl carrier protein in *Escherichia coli*. We engineered ribozymes with varying catalytic cores and arm lengths, finding that while short-armed ribozymes showed higher activity *in vitro*, long-armed variants demonstrated superior growth inhibition *in vivo*. Isothermal titration calorimetry confirmed tight binding between the ribozymes and the mRNA substrate, with association constants between 10^7^ and 10^8^ M^−1^, and gel electrophoresis verified substrate cleavage. Ribozymes were incorporated into bacterial plasmids, introduced via transformation into *E. coli*, and were expressed in a controlled manner, inhibiting bacterial growth by up to 70% over 24 h. Notably, ribozymes embedded within tRNA structures, a strategy intended to protect them from intracellular degradation, showed differential effectiveness compared to standalone variants; tRNA scaffolding preserved activity in long-armed but abolished it in short-armed constructs. Growth inhibition resulted from both mRNA cleavage and translational blocking, as demonstrated by comparing active ribozymes with their catalytically inactive variants. Furthermore, tetracycline efficacy was enhanced 2- to 4-fold in cells expressing ribozymes, indicating potential for synergy. This study demonstrates the first successful targeting of an essential gene in *E. coli* using hammerhead ribozymes, achieving growth inhibition through combined mechanisms of mRNA blocking and cleavage, and highlighting the potential of ribozymes as antibacterial strategies.

## Graphical abstract

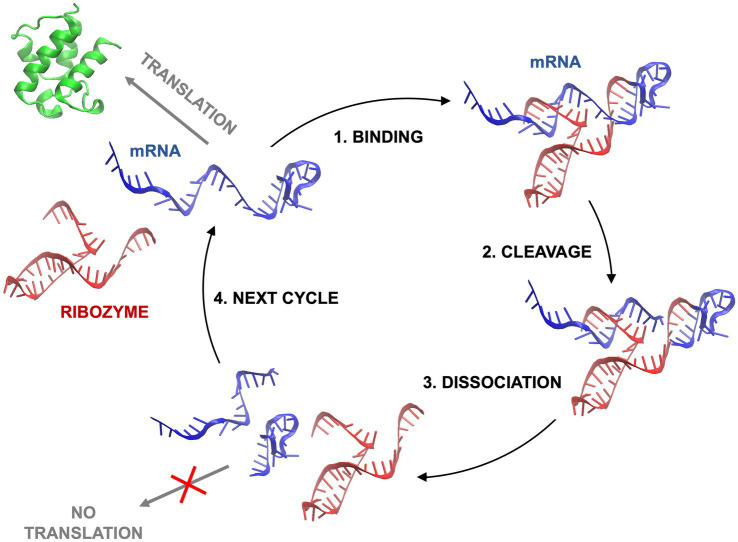

## 1 Introduction

Antimicrobial resistance (AMR) represents a global crisis, particularly with the increasing number of antibiotic-insensitive bacterial strains. It is estimated that by 2050, diseases caused by drug-resistant microbes will lead to 10 million deaths annually (O’Neill, 2014). In 2019 alone, approximately 1.3 million deaths were directly caused by antibiotic-resistant bacteria, while about 5 million deaths were associated with AMR. These infections caused about 1.5 times more deaths than human immunodeficiency virus (HIV) and twice as many as malaria. Notably, *Escherichia coli* (family *Enterobacteriaceae*) was identified as the most lethal bacterial species among all resistant pathogens (Murray et al., 2022), which is concerning given that *E. coli* normally serves as a key commensal organism within the human microbiota. While typically harmless, *E. coli* can become a significant or even life-threatening health hazard if the host’s intestinal barriers are compromised or immunity weakens (Kaper et al., 2004).

*E. coli*, commonly found in the human gut (Mueller et al., 2015), maintains microbiome balance and protects the host against pathogens (Richter et al., 2018). However, commensal *E. coli* has evolved into several pathogenic variants. The well-characterized *E. coli* virulence pathotypes associated with the intestinal tract include: diffusely adherent—DAEC, enteroaggregative—EAEC, enteroinvasive—EIEC, enterohaemorrhagic—EHEC, enteropathogenic—EPEC, and enterotoxigenic—ETEC strains. Extra-intestinal pathogenic strains include meningitis-associated *E. coli* (MNEC) and uropathogenic *E. coli* (UPEC) (Kaper et al., 2004; Pakbin et al., 2021).

The challenge in treating infections caused by these strains stems from their increasing AMR. The most commonly used antibiotics, β-lactams, encompass penicillins, cephalosporins, and their derivatives—carbapenems and monobactams. Pathogenic bacteria have developed resistance to all classes of β-lactams through several mechanisms: (i) efflux of these compounds from the periplasmic space via specific pumps, (ii) decreased penetration to the target site, and, most importantly, (iii) production of β-lactamases, enzymes that inactivate the antibiotics by hydrolyzing their β-lactam ring. A prevalent resistance mechanism involves the production of extended-spectrum β-lactamases (ESBLs) that effectively hydrolyze most β-lactam antibiotics. Pathogenic *E. coli* strains are among the principal ESBL producers (Bush and Bradford, 2016; Lima et al., 2020). Consequently, research focused on new approaches for inhibiting bacterial growth and combating bacterial resistance is crucial, particularly for *E. coli*, which continuously evolves into new infectious strains (Cantón et al., 2008; Hrabák et al., 2009).

Given the growing challenge of antibiotic resistance in *E. coli* pathogens, there is an urgent need to explore alternative strategies with mechanisms distinct from conventional antibiotics. Nucleic acid-based antimicrobials targeting specific bacterial genes have shown promise. In particular, ribozymes—RNA molecules with catalytic activity—represent an intriguing possibility for developing sequence-specific agents that could selectively target and cleave essential bacterial transcripts, inhibiting bacterial growth through mechanisms different from traditional antibiotics.

Ribozymes are RNA enzymes that catalyze a variety of biochemical reactions, including 2′,3′-cyclic phosphate hydrolysis, RNA cleavage, ligation, phosphorylation, capping, polymerization, amino acid adenylation, cofactor synthesis, aminoacylation, and acyl transfer (Silverman, 2008). Naturally occurring ribozymes exist in the genomes of a wide range of organisms, including primates and other mammals, rodents, birds, reptiles, amphibians, fish, insects, parasites, fungi, bacteria, and viruses (Seehafer et al., 2011). Ribozymes vary significantly in length, from 30 to 3,000 nucleotides, and in structure (Doudna and Cech, 2002; Tanner, 1999). Metal ions, especially magnesium, are often necessary to stabilize the functional tertiary structure of ribozymes (Inoue et al., 2004; Orita et al., 1995).

Most ribozymes cleave RNA *in cis*, within the same molecule, through a process known as self-cleaving or self-splicing (Peng et al., 2021; Weinberg et al., 2019). However, their catalytic potential has been exploited to create *trans*-cleaving ribozyme versions that target and cleave other RNA molecules. After cleavage, a ribozyme can process the next substrate and repeat the process (Carbonell et al., 2011; Pavco et al., 2000). Consequently, this *trans*-cleaving capability has been successfully tested in clinical trials against certain diseases and proposed as a potential gene therapy strategy. For instance, ANGIOZYME^®^ has shown anti-tumor effects in animal studies (Pavco et al., 2000; Weng and Usman, 2001). This anti-angiogenic ribozyme targets the mRNA of the vascular endothelial growth factor receptor, which plays a role in tumor angiogenesis and metastasis. Human clinical trials for patients with advanced malignancies are underway, showing good tolerance and absence of significant adverse effects. HERZYM^®^, another ribozyme, has undergone clinical trials for breast and ovarian cancer treatment. This RNA enzyme targets the mRNA of the human epidermal growth factor, a proto-oncogene involved in DNA repair, drug resistance, tumorigenesis, and metastasis (He et al., 2010).

Ribozyme activity has been used to prevent protein overexpression in genetic diseases including oculopharyngeal muscular dystrophy (Kharma et al., 2016), myotonic dystrophy type 1 (Langlois et al., 2003), Alzheimer’s (Nawrot et al., 2003) and Parkinson’s diseases (Hayashita-Kinoh et al., 2006), as well as in treating glioblastomas (Grzelinski et al., 2009). RNA enzymes have also shown activity against viruses such as herpes (Trang, 2001), hepatitis viruses [HBV (Weinberg et al., 2000), HCV (Welch et al., 1996)], influenza A (Kumar et al., 2012), HIV (Scarborough and Gatignol, 2015), and SARS (Fukushima et al., 2009). Some studies have demonstrated ribozyme activity against drug-resistance genes, in metabolic modulation, as anti-glucokinase agents in a diabetic animal model (Kijima, 1995), and in repairing RNA mutations (Kong, 2003).

**Figure 1:**
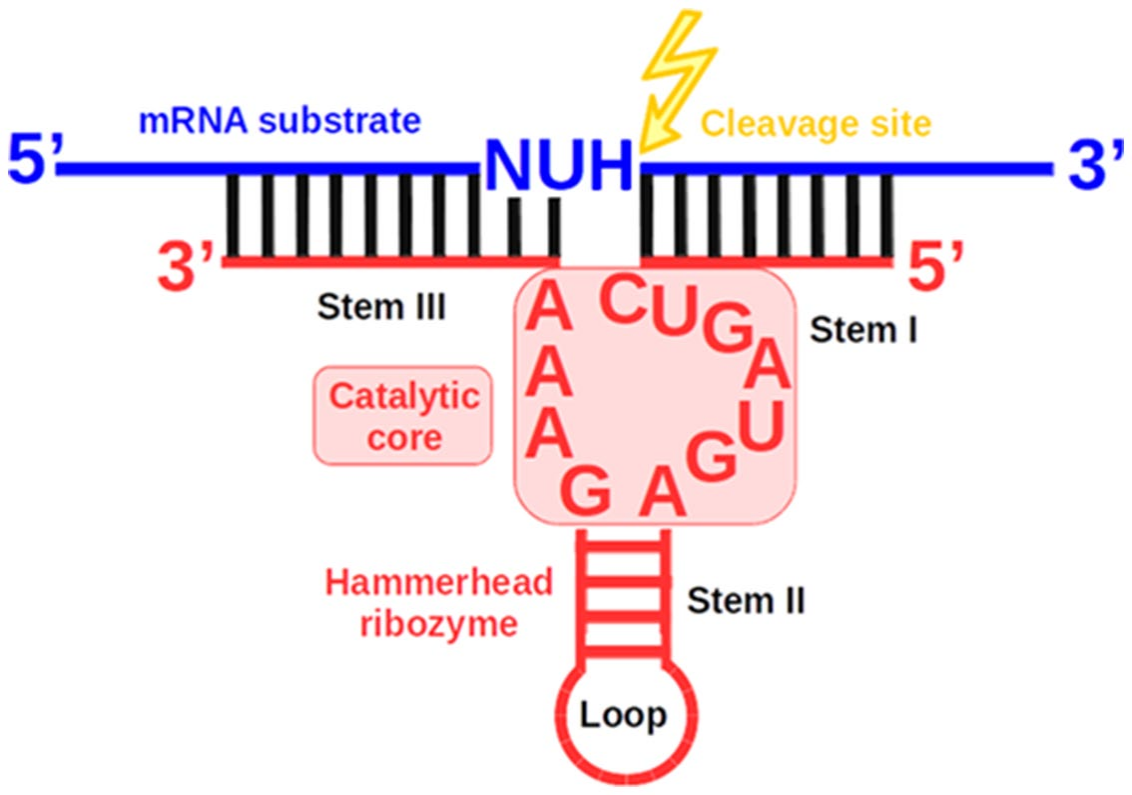
Schematic of the complex formed between a *trans*-cleaving hammerhead ribozyme (Rz) and its mRNA substrate. The structure consists of Stem II and a loop, alongside Stems I and III, which are formed by Watson-Crick base pairing between the two Rz arms and the mRNA substrate.

Among these reports, all but two employed the naturally occurring hammerhead ribozyme (Rz), the smallest and best-characterized model ribozyme. Rz operates as a *cis*-acting enzyme and consists of three stems and two or three loops (Perreault et al., 2011). Rz catalyzes a transesterification reaction yielding two cleavage products: the 5′ product with a 2′,3′-cyclic phosphate at its 3′ terminus and the 3′ product with a hydroxyl group at its 5′ terminus (Bratty et al., 1993; Hammann et al., 2012). The *cis*-cleaving Rz can be engineered into a *trans*-cleaving variant that forms a complex with its target substrate as a separate strand (Figure 1) (Carbonell et al., 2012). However, a conserved catalytic core, important for Rz activity, imposes constraints on the sequence of the RNA substrate. The target’s cleavage site must be located directly after the nucleotide triplet 5’-NUH↓-3′ (N—any nucleotide, U—uracil, H—any nucleotide except guanine) (Kore, 1998). The specific activity of Rz and the cleavage site sequence requirements enable the transesterification reaction to be carried out at precisely defined locations on mRNA substrates. Moreover, the small size of Rz makes it potentially easier to deliver into cells.

The catalytic core of Rz has been subjected to various modifications such as adding extra residues (De la Peña and Flores, 2001; Thomson et al., 1996), substitutions (Dawson and Marini, 2000; Myers and Sullivan, 2024; Wang et al., 1999), or deletions (Lott et al., 1998). Most variants showed lower efficiency. The replacement of three adjacent adenosines in the catalytic core with three uracils resulted in ribozyme inactivation (Figure 1) (Naghdi et al., 2020). Substitution of the first or third adenine of the three adjacent adenines in the catalytic core significantly decreased the rate of substrate cleavage (Ruffner et al., 1990; Slim and Gait, 1992). In contrast, other studies reported that even in the absence of the three core adenines, cleavage was still efficient (Lott et al., 1998). Additional studies have described modifications that improve cleavage capability (De la Peña and Flores, 2001). Nevertheless, new and more efficient catalytic core sequences are still being sought.

The Rz (*trans-*acting variant) is the most commonly used therapeutic ribozyme, tested against tumors (He et al., 2010; Pavco et al., 2000; Weng and Usman, 2001), viruses (Fukushima et al., 2009; Kumar et al., 2012; Scarborough and Gatignol, 2015; Weinberg et al., 2000), for repairing RNA mutations (Kong, 2003), and in treating neurological (Grzelinski et al., 2009; Hayashita-Kinoh et al., 2006; Nawrot et al., 2003) and muscular diseases (Kharma et al., 2016; Langlois et al., 2003). Despite its potential, studies on ribozymes for bacterial mRNA cleavage, particularly in pathogenic strains, are limited. For example, Yadava et al. reported on an Rz targeting the mRNA encoding the transcriptional repressor *lexA*, which was expressed under IPTG induction in *E. coli* BL21(DE3). Although the Rz’s catalytic activity was observed, *lexA* is not an essential gene, which is required to achieve bacterial growth inhibition (Yadava et al., 2005). In another study, an Rz targeting the non-essential *lacZ’* gene successfully reduced *lacZ’* expression in *E. coli* XL1-Blue transfected with a phage vector (Junn and Kang, 1996). Moreover, bacterial growth was inhibited by cleaving the mRNA of the essential *ftsZ* gene (responsible for bacterial cell division) with a DNA enzyme (Tan et al., 2004). Additionally, an RNase P-based ribozyme effectively cleaved the mRNA of a proto-oncogene in bacterial and mammalian cells. In this case, both the ribozyme and the proto-oncogene were introduced into the cells via plasmids (Toumpeki et al., 2018). In another study, a delta ribozyme targeted the bacterial mRNA of a gene that is considered essential, but the resulting reduction in gene expression was insufficient to inhibit growth. Furthermore, this study used the avirulent Gram-positive *Lactococcus lactis* strain (Fiola et al., 2006). Although different in their approaches, these studies provide the foundation for using Rz to directly target essential genes in pathogenic bacteria.

To our knowledge, no studies have investigated ribozyme activity upon directly targeting the mRNA of essential bacterial genes in pathogenic strains. An essential gene in many bacteria is *acpP*, which encodes the acyl carrier protein (ACP) (Castillo et al., 2018; Dryselius et al., 2003; Good et al., 2001). ACP is a highly conserved protein of 77 amino acids (Worsham et al., 2003) and plays a fundamental role in *E. coli* metabolism as a crucial carrier and cofactor in the biosynthesis of fatty acids (Chan and Vogel, 2010; Jackowski and Rock, 1987), lipopolysaccharides (Gronow and Brade, 2001), and phospholipids (Cronan and Rock, 2008). Importantly, ACP enables pathogenic *E. coli* to convert the non-toxic prohemolysin to hemolysin, a mature toxin that destroys mammalian cells by forming pores (Issartel et al., 1991). The *acpP* transcript is an effective target for antisense inhibition using peptide nucleic acid (PNA) oligomers (Castillo et al., 2018; Dryselius et al., 2003; Good et al., 2001; Hatamoto et al., 2010; Popella et al., 2022; Równicki et al., 2019). Bacterial growth inhibition was observed upon targeting *acpP* mRNA with low μM concentrations of PNA_anti-*acpP*_ conjugated with a (KFF)_3_K peptide or vitamin B_12_ (Równicki et al., 2019).

In this study, we report a ribozyme-based approach to target the *acpP* mRNA transcript encoding ACP in *E. coli*. We hypothesized that inhibiting ACP expression by introducing a ribozyme specifically targeting *acpP* mRNA into *E. coli* would inhibit bacterial growth. We designed four Rz variants, tested their binding with the *acpP* mRNA fragment, and confirmed *in vitro* cleavage of this mRNA substrate. We then constructed plasmids encoding the designed Rz variants, introduced them into *E. coli* via transformation, and examined bacterial growth under IPTG-controlled expression of the ribozymes.

## 2 Materials and methods

### 2.1 *In silico* predictions of the substrate-ribozyme complexes

*In silico* two-dimensional substrate-Rz complexes were predicted using the Bimolecular Structure Prediction by RNAstructure 6.4 (Mathews Lab, University of Rochester, NY, United States) (Bellaousov et al., 2013). Three-dimensional structures were modeled using the RNAfold Web Server (Gruber et al., 2008) and RNAComposer (Popenda et al., 2012) and visualized with the Visual Molecular Dynamics program (VMD) (Humphrey et al., 1996).

### 2.2 Plasmids, RNA, and DNA oligonucleotides

The RNA oligonucleotides, including the *acpP* mRNA fragment as a substrate (labeled mRNA*_acpP_*) and the ribozymes, long-armed (Rz_long_) and short-armed (Rz_short_), were purchased from Future Synthesis (Poland, Poznań). Plasmids based on pUC57-Kan (cloning vector with a kanamycin resistance marker; 2,579 bp; *ori* pMB1) containing the Rz sequences, designed using SnapGene software (www.snapgene.com), were purchased from BioCat (Germany, Heidelberg). Plasmid DNA and RNA oligonucleotides were dissolved in autoclaved Milli-Q (MQ) water. Sample concentrations were measured using a DeNovix DS-11 spectrophotometer.

### 2.3 Bacterial strains and media components

The *E. coli* strains used were BL21(DE3), which encodes T7 RNA polymerase (Jeong et al., 2009), and TOP10, which has high transformation efficiency (Yang and Yang, 2012; Liu et al., 2018). The following media and reagents were used for bacterial cultures: Lysogeny Broth—LB (VWR), Lysogeny Broth with Agar—LA (VWR), Mueller Hinton Broth—MHB (Difco), Davis Minimal Medium (Sigma) with a final concentration of 0.4% D-(+)-glucose (Sigma), kanamycin—Kan (Gibco, Fluorochem), magnesium chloride—MgCl_2_ (Sigma), calcium chloride—CaCl_2_ (Sigma), isopropyl β-d-1-thiogalactopyranoside—IPTG (BioShop), glycerol (Avantor), and ultra-pure MQ water—Integral 10. Growth media, 0.1 M CaCl_2_, and 50% glycerol, were autoclaved. Stock solutions of D-(+)-20% glucose, 50 mg/mL Kan, 1 M MgCl_2_, and 1 M IPTG were sterilized using sterile PES syringe filters with a 0.22 μm pore size (GenoPlast Biotech).

### 2.4 Isothermal titration calorimetry

*In vitro* formation of Rz and mRNA substrate complexes was monitored by isothermal titration calorimetry (ITC) using a Nano ITC (TA Instruments). RNAs were dissolved in 50 mM Tris–HCl pH 7.5 (Tris(hydroxymethyl)aminomethane/Tris—Merck, Hydrochloric acid/HCl—Honeywell), 10 mM MgCl_2_ (Sigma), and incubated separately at 37 °C for 10 min outside the calorimeter. The final RNA concentrations and ratios were: substrate: Rz_short_ (32 μM:7 μM) and substrate: Rz_long_ (42 μM:6 μM). Then, an excess of mRNA substrate dilution of 70 μL was placed in the titration syringe and the ribozyme dilution of 300 μL was placed in the calorimeter sample cell. The reference cell was filled with MQ water. The system was equilibrated for 200 s to establish a stable baseline before the first injection. Next, 17 injections of 3 μL of the mRNA substrate were titrated every 500 s into the sample cell containing Rz and stirred at 350 rpm. Data were analyzed using NanoAnalyze software (TA Instruments).

### 2.5 Cleavage reaction and gel electrophoresis

*In vitro* cleavage reactions were performed in the buffer (50 mM Tris–HCl pH 7.5, 10 mM MgCl_2_). The mRNA substrate and ribozymes were first denatured separately for 1 min at 90 °C. Next, the samples were cooled to 37 °C to allow refolding of tertiary structures. The reactions were then initiated by mixing the RNAs and incubating at 37 °C. Samples of the substrate and Rz complexes were collected over time and the reaction was stopped by adding 50 mM EDTA (ThermoFisher Scientific). Next, the samples were stabilized with formamide (FORMAzol, Molecular Research Center) in the 1:1 RNA: FORMAzol ratio. Control samples, containing either the substrate or Rz alone, were loaded with 6x Loading Dye (ThermoFisher Scientific). Before loading, the RNA samples were denatured for 3 min at 55 °C (short-armed Rz) and 55 °C or 70 °C (long-armed Rz). Substrate cleavage was analyzed by separating the samples on 15, 18% or 20% denaturing polyacrylamide gels with 7 M or 8 M urea, 10% APS, and TEMED (40% Acrylamide-Bis Solution—Bio-Rad, urea—Sigma, *Ammonium Persulfate/APS—Sigma,* N, N, N′, N′-Tetramethylethylenediamine/TEMED—Sigma) in 1xTBE buffer (Tris—Merck, Boric Acid—Sigma, Disodium ethylenediaminetetraacetate dihydrate/Na_2_EDTA—Sigma) using a vertical gel electrophoresis system (ASG-250 Gel System, C. B. S. Scientific). After the gel polymerized, the wells were rinsed and a pre-run electrophoresis without samples was performed in 1xTBE running buffer for 30 min at 400 V. The wells were then rinsed again, samples were loaded, and the main run was performed for 3 h 30–45 min at 400 V. After electrophoresis, gels were stained with SYBR Gold (Invitrogen, ThermoFisher Scientific) dissolved in 1xTBE. Gels were imaged using a Molecular Imager Gel Doc XR + system (Bio-Rad) and the band intensities were measured using the Image Lab Software (Bio-Rad).

### 2.6 Preparation of *Escherichia coli* competent cells

Competent cells of *E. coli* TOP10 were prepared to store plasmids as reserve stocks in the bacterial bank using the *E. coli* Transformer Kit (A&A Biotechnology). Competent cells of *E. coli* BL21(DE3) were prepared as follows. The strain was plated on LA and incubated overnight at 37 °C. A single bacterial colony was inoculated into 10 mL of LB medium and incubated overnight at 37 °C with shaking at 600 rpm (Thermomixer comfort, Eppendorf). 1 mL of the overnight culture was added to 100 mL of fresh LB and incubated at 37 °C with shaking at 165 rpm (Incubator Shaker Series, Innova 44) until the culture reached optical density at 600 nm (OD_600_) between 0.2 and 0.3. The culture was divided into two 50 mL sterile falcon tubes and incubated on ice for 20 min. The cells were then centrifuged for 15 min at 4 °C at 5,000 × g (Centrifuge 5810 R, Eppendorf). Supernatants were removed and each pellet was resuspended in 10 mL of ice-cold 0.1 M CaCl_2_, incubated on ice for 40 min, and again centrifuged for 15 min at 4 °C at 5,000 × g (Centrifuge 5810 R, Eppendorf). The supernatants were removed and each pellet was resuspended in 0.6 mL of ice-cold 0.1 M CaCl_2_. Next, 0.6 mL of 50% ice-cold glycerol was added. The cells were mixed, aliquoted into 100 μL portions, and stored at −80 °C.

### 2.7 Transformation of plasmid DNA into *Escherichia coli* strains

*E. coli* TOP10 competent cells were transformed with plasmids according to the *E. coli* Transformer Kit (A&A Biotechnology). Plasmid DNA containing mutated inactive ribozymes was transformed into high-efficiency NEB 5-alpha competent *E. coli* cells, provided with the Q5^®^ Site-Directed Mutagenesis Kit, following the manufacturer’s protocol. *E. coli* BL21(DE3) competent cells were transformed using the heat-shock method. Briefly, aliquots of *E. coli* BL21(DE3) competent cells and plasmids were thawed on ice for 15 min. Fifty nanogram of plasmid DNA was then added to each 100 μL aliquot of competent cells and stirred gently. A no-DNA control sample with sterile water was always transformed to monitor for contamination. The mixtures were incubated on ice for 30 min. Next, the competent cells were heat-shocked at 42 °C for 2 min and placed on ice for an additional 2 min. After that, 1 mL of LB medium (pre-heated to 37 °C) was added to each mixture and incubated at 37 °C for 1 h with gentle shaking at 300 rpm (Thermomixer comfort, Eppendorf). Subsequently, the cells were centrifuged for 1 min at 5000 rpm (Centrifuge 5415 R, Eppendorf). One milliliter of LB was removed and the bacterial pellet was resuspended in the remaining volume (100 μL) of the medium. Next, the cells were plated on LA plates containing Kan and then incubated overnight at 37 °C.

### 2.8 Bacterial cultures for *in vivo* cleavage with induced ribozymes

A single bacterial colony was inoculated into a 1 mL of medium (MHB, Davis). Overnight cultures in MHB were supplemented with Kan (final concentration of 50 μg/μL) and were prepared either with or without Mg^2+^, while cultures in Davis medium contained neither Kan nor Mg^2+^. The bacteria were incubated overnight at 37 °C with shaking. The next day, experiments were performed using either glass falcon tubes or 96-well microplates.

For the experiments with falcon tubes, each overnight culture was diluted 100x in 2 mL of the same fresh medium. MHB cultures were supplemented with or without Mg^2+^ (final concentrations: 2.5, 5, or 10 mM). During incubation at 37 °C with shaking at 600 rpm, OD at 600 nm was measured every 30 min or 60 min using a spectrophotometer SP-830 Plus Metertech. After 2.5 h of growth for MHB and 4 h for Davis medium, ribozyme expression was induced by adding IPTG to a final concentration of 1 mM. Each reading was preceded by a blank measurement with clear unsupplemented medium, so the raw data could be used directly in the graphs.

For experiments in microplates, overnight cultures were diluted to the identical final dilution of 100x in a fresh medium to a final volume of 100 μL per well. For each biological replicate, each culture condition had 6 technical replicates, grown with or without 1 mM IPTG. IPTG was added at the beginning of the experiment, prior to adding bacteria. Two independent biological experiments were performed in MHB and two or three in Davis medium. Twenty-four wells containing clear unsupplemented medium were used for background subtraction and as a sterility control. Each microplate was sealed with a transparent sterile film (Microplate Sealing Films, EXCEL Scientific). OD at 600 nm was measured using a microplate reader (Biotek Synergy H1) every 30 min using Gen5 software. The sterility control readings were subtracted from the OD of the bacterial culture. Data from technical replicates for every culture were averaged and analyzed using a two-way ANOVA test. Graphs were prepared with GraphPad Prism 8 software. A *p*-value < 0.05 was considered statistically significant.

### 2.9 Monitoring the potentiation of ribozyme with tetracycline on inhibition of *Escherichia coli* growth

The 20-h growth kinetics of bacterial cultures containing different Rz plasmids, the control plasmid without Rz, and a no-plasmid control, were monitored in MHB medium incubated with tetracycline and with 1 mM IPTG using the microplate method described above. Tetracycline concentrations of 16, 8, 4, 2, 1, or 0.5 μM were prepared following the broth microdilution protocol of the Clinical and Laboratory Standards Institute method M07-A10. Experiments were performed in two biological replicates, each with 2–5 technical replicates.

## 3 Results

### 3.1 Selection of the target sequence of the *acpP* mRNA substrate and design of Rz variants

**Figure 2:**
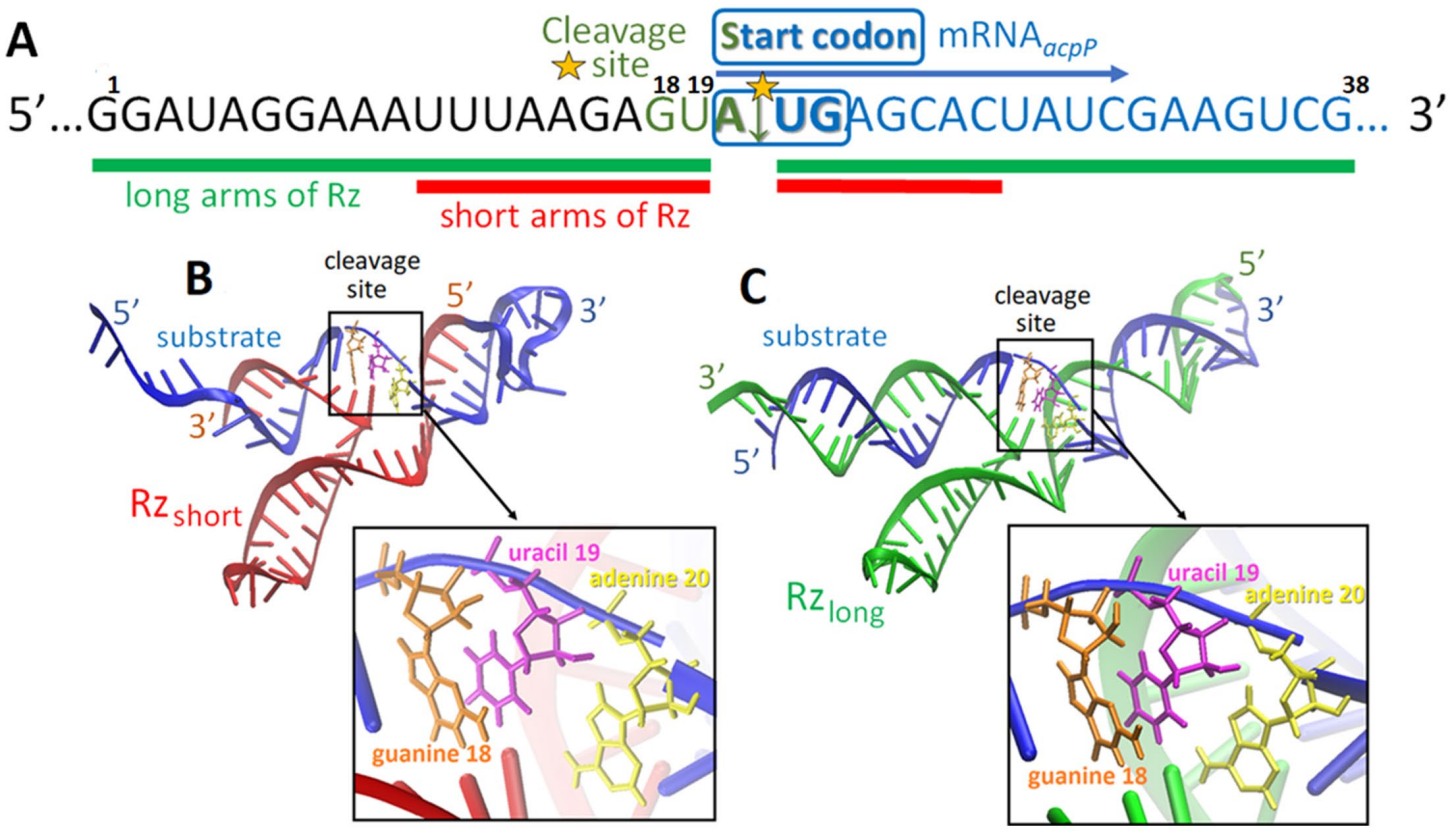
The target sequence surrounding the predicted cleavage site of the mRNA*_acpP_* transcript **(A)**. Predicted tertiary structures of the ribozyme complexes between the substrate and Rz_short_ **(B)** and the substrate and Rz_long_ **(C)**. The yellow star in **(A)** indicates the cleavage site.

Previous studies (Dryselius et al., 2003; Good et al., 2001), including ours (Castillo et al., 2018; Równicki et al., 2019), showed that blocking the GAGUAUGAG region of the *acpP* mRNA, which encompasses the start codon (underlined), with a complementary peptide nucleic acid oligomer, leads to the inhibition of ACP expression and subsequent *E. coli* growth. Within this *acpP* mRNA sequence, the nucleotide triplet motif (5′-NUX-3′) at which Rz can cleave is present. Therefore, we selected the GUA sequence in the *acpP* mRNA as the most likely position for efficient cleavage by Rz (Kore, 1998). We designed two Rz variants differing in the length of their hybridization arms and targeted to the mRNA*_acpP_* substrate cleavage site: Rz_short_ with 7- and 9-nucleotide-long arms, and Rz_long_ with two 19-nucleotide arms (Table 1; Figure 2A). For each variant, the Rz catalytic core was either kept in its original form or was extended by one adenine based on *in silico* predictions suggesting improved binding affinity of the mRNA substrate with the ribozyme. As such an extended catalytic core had not been previously tested, we decided to explore both possibilities.

**Table 1:** Sequences of RNA oligonucleotides.

Molecule	RNA sequence 5′→3′	Length [nt]
mRNA substrate (mRNA*_acpP_*)	GGAUAGGAAAUUUAAGA *GUA↓* UGAGCACUAUCGAAGUCG	38
Short ribozyme (Rz_short_)	GUGCUCA**CUGAUGA**GGCCGAAAGGCC**GAAAA**CUCUUAAA	39
Long ribozyme (Rz_long_)	GCGACUUCGAUAGUGCUCA**CUGAUGA**GGCCGAAAGGCC**GAAAA**CUCUUAAAUUUCCUAUCG	61

The cleavage site within the mRNA substrate is italicized and underscored. The catalytic core of the ribozyme is in bold, with the extra A underlined.

Alignment of the mRNA*_acpP_* sequences of the *E. coli* BL21(DE3) strain used in this study with those of selected pathogenic *E. coli* strains revealed 100% similarity across the target region (Supplementary Table S1). This result underscores the potential for using the mRNA encoding the ACP protein as a potent ribozyme target in pathogenic strains as well.

*In silico* methods were used to generate the tertiary structure models of the complexes of Rz_long_ and Rz_short_ with the mRNA substrate (Figures 2B,C). These models illustrate the Rz arms hybridizing with the mRNA substrate at predicted positions flanking the start codon.

**Figure 3:**
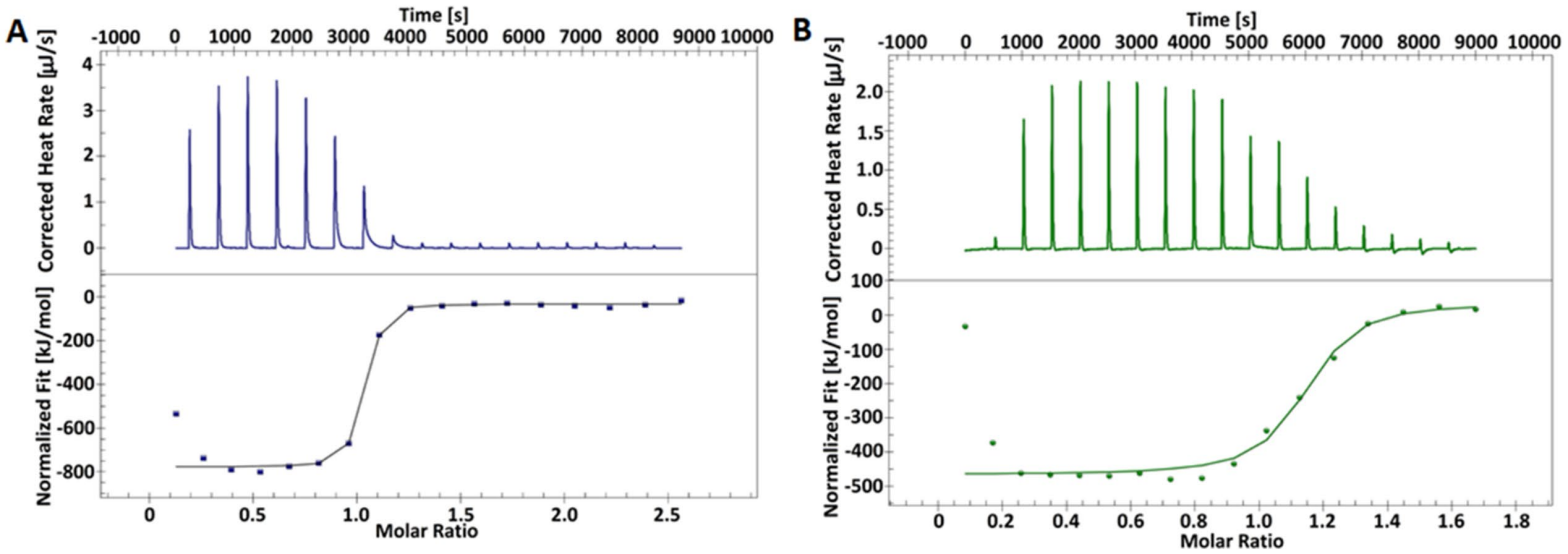
Representative ITC thermograms from the titration of the mRNA substrate into Rz_long_ **(A)** and Rz_short_ **(B)** ribozymes. The top panels show the raw heat data for each injection, and the bottom panels show the integrated heat plotted against the molar ratio of the substrate to Rz. The solid line represents the best fit to a 1:1 binding model.

Next, we used ITC to characterize binding between ribozymes and the mRNA*_acpP_* substrate. Titration of the substrate into the ribozymes yielded sigmoidal binding isotherms, indicating an exothermic and spontaneous binding process (Figure 3). The mRNA substrate and Rz_short_ form a complex with a 16-base pair (bp) interaction region, while the Rz_long_ complex involves a 38 bp interaction, as predicted *in silico* (Figure 2A). For both complexes, the data fit to a 1:1 binding model, with association constants (K_a_) in the range of 10^7^–10^8^ M^−1^, confirming the formation of stable complexes. The calculated dissociation constants (K_d_) indicate that the complex with Rz_long_ is more tightly bound than the one with Rz_short_ (Table 2). Thermodynamic parameters obtained in this study are comparable to previously reported values obtained for similar 15 bp (Mikulecky and Feig, 2004) and 23 bp Rz-substrate complexes (Reymond et al., 2009).

**Table 2:** Thermodynamic parameters for the binding of the mRNA substrate to the ribozymes, as determined by ITC.

Complex with	K_a_ [M^−1^]	K_d_ [M]	Molar ratio	ΔH [kJ/mol]	TΔS [kJ/mol]	ΔG [kJ/mol]
Substrate: Rz_short_	2.95 × 10^7^	3.39 × 10^−8^	1.1	−501.6	−457.2	−44.4
Substrate: Rz_long_	1.97 × 10^8^	5.07 × 10^−9^	1.0	−743.6	−694.4	−49.2

For RNA sequences refer, to Table 1.

### 3.2 *In vitro* cleavage of the mRNA*_acpP_* substrate by ribozymes

**Figure 4:**
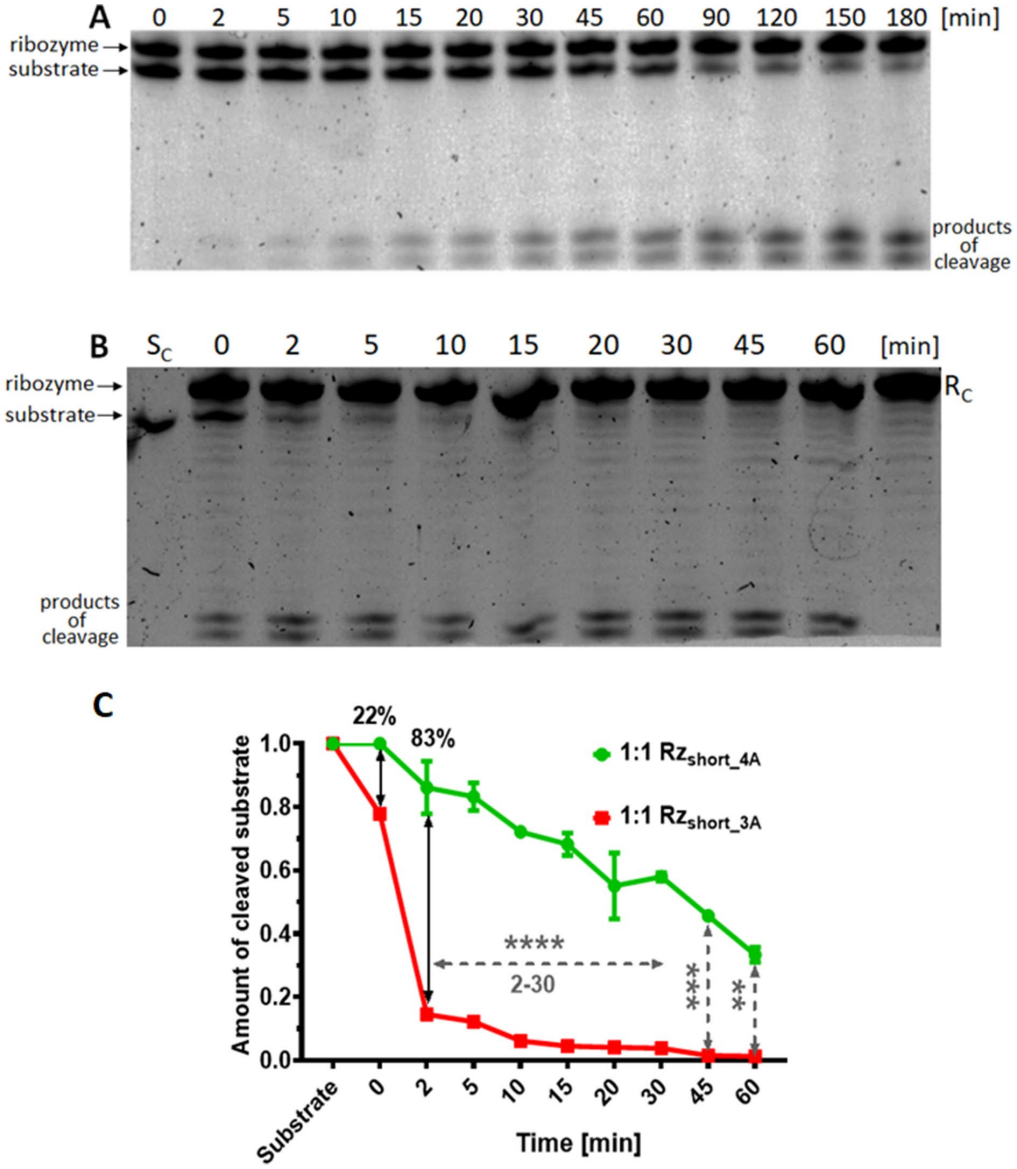
*In vitro* cleavage of the mRNA*_acpP_* substrate by Rz_short_ variants. Representative 15% denaturing polyacrylamide gels (with 7 M urea) showing the kinetics of substrate cleavage over time (0–60 min or 0–180 min) by **(A)** Rz_short_4A_ or **(B)** Rz_short_3A_ (denoted ribozyme in the figure). Reactions were performed at a 1:1 molar ratio. The standard ribozyme concentration was 2 μM. Controls: S_C_, substrate alone; R_C_, ribozyme alone. **(C)** Quantification of the mRNA*_acpP_* substrate cleavage by Rz_short_4A_ (green) and Rz_short_3A_ (red) at a 1:1 ratio after a 3-h incubation. Data are normalized and presented as the mean ± SEM (*n* = 2 for Rz_short_4A_; *n* = 1 for Rz_short_3A_). Statistical significance is indicated as follows: ****p < 0.0001, ***p < 0.001, and **p < 0.01; other differences are not significant.

Next, we tested the *in vitro* cleavage of the mRNA substrate with Rz ribozymes. Analysis of the polyacrylamide gels revealed cleavage products for both the short- and long-armed Rz variants. However, under all tested conditions, Rz_short_ was consistently faster and more efficient at cleaving the mRNA substrate than Rz_long_ (Figure 4; Supplementary Figure S1). Attempts to optimize the Rz_long_ reaction conditions, e.g., by varying incubation temperature or substrate:ribozyme ratio, did not improve the cleavage efficiency to the level exhibited by Rz_short_ (Supplementary Figure S1). The higher *in vitro* cleavage efficiency observed for Rz_short_ compared to Rz_long_ is likely due to the tighter binding between Rz_long_ and the substrate (Table 2), which could hinder the dissociation of cleavage products from Rz_long_, thereby reducing the rate of enzyme turnover, and making the cleavage of Rz_long_ less detectable (Supplementary Figure S1).

Based on the higher activity of the Rz_short_ variants, evidenced by more intense product bands, we further characterized their cleavage kinetics. As anticipated, we observed two distinct cleavage products, the appearance of which corresponded with a time-dependent decrease in the full-length substrate (Figures 4A,B). The gels showing cleavage at different molar ratios of the substrate and Rz_short_ are shown in Supplementary Figure S2. Analysis of band intensities at different ribozyme to substrate ratios indicated that substrate cleavage was most efficient when the substrate and Rz_short_ were present in equal molar quantities (Supplementary Figure S3). An excess of Rz_short_ did not improve cleavage efficiency, corroborating the 1:1 stoichiometry determined by ITC.

A comparison of the *in vitro* activity of Rz_short_3A_, which contains the three-adenosine core, with the activity of the Rz_short_4A_ variant, which contains an extra adenine, showed greater product formation and faster disappearance of the substrate band for Rz_short_3A_ (Figure 4). Specifically, Rz_short_3A_ achieved complete substrate cleavage and elimination within 45–60 min, while Rz_short___4A_ cleaved approximately 75% of the substrate in the same time frame (Figure 4C). Nevertheless, Rz_short_4A_ also cleaved the RNA substrate completely after 180 min at a 1:1 ratio (Supplementary Figure S3).

### 3.3 Design of cassettes containing the ribozyme sequences on the pUC57-Kan plasmid

To express Rz in bacteria for *in vivo* testing, we designed four cassettes encoding different Rz variants within the pUC57-Kan plasmid (Supplementary Table S2). Each cassette contains a T7 promoter and a transcription terminator for the phage T7 RNA polymerase, as well as additional strong terminators to prevent the synthesis of the ribozyme by other bacterial polymerases (Supplementary Figure S4). These flanking terminators, ilvBN and ECK120016882 (indicated as “terminator_for” and “terminator_rev*” in Supplementary Table S2) are naturally occurring (Chen et al., 2013). The main component of the cassette is the Rz, with two different arm lengths (Rz_short_ or Rz_long_) specifically tailored to hybridize with the mRNA*_acpP_* (Table 1; Figure 2). Additionally, the Rz variants are either standalone or integrated within a tRNA scaffold to protect them from enzymatic degradation [Rz_short_(tRNA) and Rz_long_(tRNA)]. An extra linker was added to the 5′-end of Rz to connect it to tRNA (Huang et al., 2019) (Supplementary Figure S5), as linkers at the 5′-end of tRNA were found to increase the effectiveness of mRNA cleavage by Rz (Medina and Joshi, 1999). The original linker from Huang et al. (2019) included an EcoRI site, so we modified its sequence to remove any restriction sites within the linker.

To optimize Rz activity *in vivo* and to be able to incorporate additional controls, we also engineered extra elements within the Rz cassette. One such element is an auto-cleaving hammerhead ribozyme, incorporated as an additional cleavage tool because of its high cleavage efficiency at 3′ termini compared to other ribozymes (Chowrira et al., 1994) (Supplementary Figure S4_1). This auto-cleaving segment cleaves at the GUC site and was positioned after the Rz or the 3’ tRNA terminus to shorten the transcript. This way, following *cis*-cleavage of the auto-cleaving segment within the cassette, only 3 extra nucleotides remain (Supplementary Figure S4_2), instead of the 48 nucleotides of the T7 terminator (Supplementary Figure S4_1). Without this self-cleaving element, Rz or Rz(tRNA) would possess an excessively long 3′-terminal tail that could interfere with forming the complex between Rz and the mRNA*_acpP_* substrate. Another element, designed as a contingency if the *acpP* transcript cleavage were to fail, is a site for inserting an additional gene. We included restriction sites (EcoRI/SpeI) for cloning the red fluorescent protein (RFP) gene, which would enable conversion of Rz into a control ribozyme that would target and cleave mRNA*_rfp_*. This arrangement would allow monitoring of cleavage of an mRNA encoded on the same plasmid by measuring RFP fluorescence (Równicki et al., 2017). The designed cassettes also contain other restriction sites (e.g., SpeI, AgeI) to facilitate fragment replacements through restriction enzyme digestion and ligation. Moreover, all sequences can be easily modified using polymerase chain reaction (PCR). Finally, to distinguish growth inhibition caused by mRNA steric blocking versus cleavage, we prepared two catalytically inactive ribozyme variants (Supplementary Figure S6).

### 3.4 Optimizing growth conditions of bacteria with ribozyme-encoding plasmids

We introduced the plasmid constructs containing the Rz variants into *E. coli* BL21(DE3). The presence of the T7 RNA polymerase gene, under the control of the *lacI* repressor and *lac* UV5 promoter, along with the T7 promoter sequence on the plasmid vector, ensures that upon successful transformation, Rz expression occurs intracellularly and can be controlled by IPTG (Supplementary Figure S7).

When culturing bacteria containing a plasmid that encodes antibiotic resistance, it is standard practice to supplement the medium with the corresponding antibiotic. However, to avoid potential interference with the ribozyme-catalyzed reaction, we chose to conduct the experiments without the antibiotic. Aminoglycoside antibiotics, including Kan, bind various RNAs, and could potentially bind Rz and inhibit its transesterification reaction (Stage et al., 1995). To determine whether Kan affected our results, we performed experiments with and without Kan, and compared bacterial growth (Supplementary Figure S8). The results showed similar OD_600_ levels for the control strain and those transformed with Rz-encoding plasmid constructs, regardless of the presence of Kan or IPTG induction. Based on these results, we conducted subsequent experiments without Kan supplementation.

Furthermore, *in vitro* studies have shown that Mg^2+^ ions are important for forming an active complex between Rz and its RNA substrate (Orita et al., 1995), with Rz catalytic activity increasing with Mg^2+^ concentration (Inoue et al., 2004). Consequently, we investigated whether *in vivo* Rz activity is affected by supplemental Mg^2+^. We supplemented the medium with 2.5, 5, and 10 mM Mg^2+^. The results showed that higher Mg^2+^ concentrations reduced growth-inhibitory effects arising from Rz_long_, even with IPTG induction (Supplementary Figure S9). Thus, contrary to *in vitro* findings (Inoue et al., 2004; Orita et al., 1995), our data showed that excess Mg^2+^ adversely affects *in vivo* Rz performance. While Mg^2+^ influences *in vitro* cleavage, *in vivo* these ions appear to support bacterial growth rather than enhance cleavage. Based on these observations, we conducted subsequent experiments without supplemental Kan or Mg^2+^.

We also monitored the effect of IPTG on the growth of bacterial cultures. We found that in MHB medium, the OD_600_ of Growth Control and Plasmid Control (containing the pUC57-Kan vector) was consistently lower (by approximately 0.1) in the presence of IPTG compared to cultures grown without IPTG (Supplementary Figures S10, S11). Therefore, the lower OD of bacteria containing Rz could not be conclusively attributed to Rz activity because it could also result from the metabolic burden imposed by IPTG addition. To address this issue, we changed the culture medium from MHB to Davis minimal medium. In Davis medium, the OD values for Growth Control with and without IPTG, were similar, as were OD values for Plasmid Control with and without IPTG (Supplementary Figures S10–S12). We therefore concluded that the reduced OD of bacteria containing Rz_long_, observed after IPTG addition, was likely due to plasmid expression and ribozyme activity.

We also monitored bacterial growth in MHB and Davis media using a microplate reader, as opposed to the above experiments performed in falcon tubes. As expected, the highest OD_600_ was observed for the Growth Control (Supplementary Figure S13). In both media without IPTG, the growth curves for *E. coli* containing plasmids with Rz_long_ and Rz_short_ showed OD_600_ values 0.1 to 0.2 lower than the Growth Control, demonstrating statistically significant growth inhibition. Importantly, after IPTG induction, both Rz_long_ and Rz_long_(tRNA) visibly inhibited bacterial growth in both MHB and Davis media (Supplementary Figure S13).

Interestingly, in experiments using MHB medium in both falcon tubes (Supplementary Figures S8–S11) and microplates (Supplementary Figure S13), we consistently observed a delayed onset of the logarithmic growth phase in bacteria containing Rz_long_ grown without IPTG. This led us to hypothesize that a component within the medium was causing T7 promoter leakage and expression of Rz_long_. The MHB medium contains starch (1.5 mg/mL = 7.14%), a saccharide similar to other saccharides present in plant-based media (Krefft et al., 2022), and structurally similar to IPTG, which may induce the expression of the *lac* promoter in *E. coli* BL21(DE3). This likely explains the unintended expression of Rz_long_ in MHB without IPTG. In contrast, glucose, which is present in Davis minimal medium, does not cause such leakage (Krefft et al., 2022).

The OD of the *E. coli* culture with the pUC57-Kan vector (Plasmid Control) was higher than that of bacteria containing a plasmid with the ribozyme cassettes, indicating that the observed growth inhibitions can be attributed to ribozyme expression. The 20- to 24-h kinetics confirmed that inhibition of bacterial growth was most pronounced in Davis medium (Supplementary Figure S14; Supplementary Table S3). We also noted a lower OD for bacterial cultures with the Plasmid Control compared to the Growth Control after 24 h (Supplementary Figure S15), likely due to the burden imposed by the plasmid (Diaz Ricci and Hernández, 2000; Ow et al., 2006). However, comparing the OD measured for the Rz_long_ and Rz_long_(tRNA) cultures to the OD of the Plasmid Control in Davis medium with IPTG (Supplementary Figure S15) shows that the reduction in bacterial growth is due to the activity of the expressed ribozymes and not merely the presence of the plasmid vector itself.

### 3.5 Antibacterial activity of the ribozyme directed to target bacterial *acpP* mRNA

**Figure 5:**
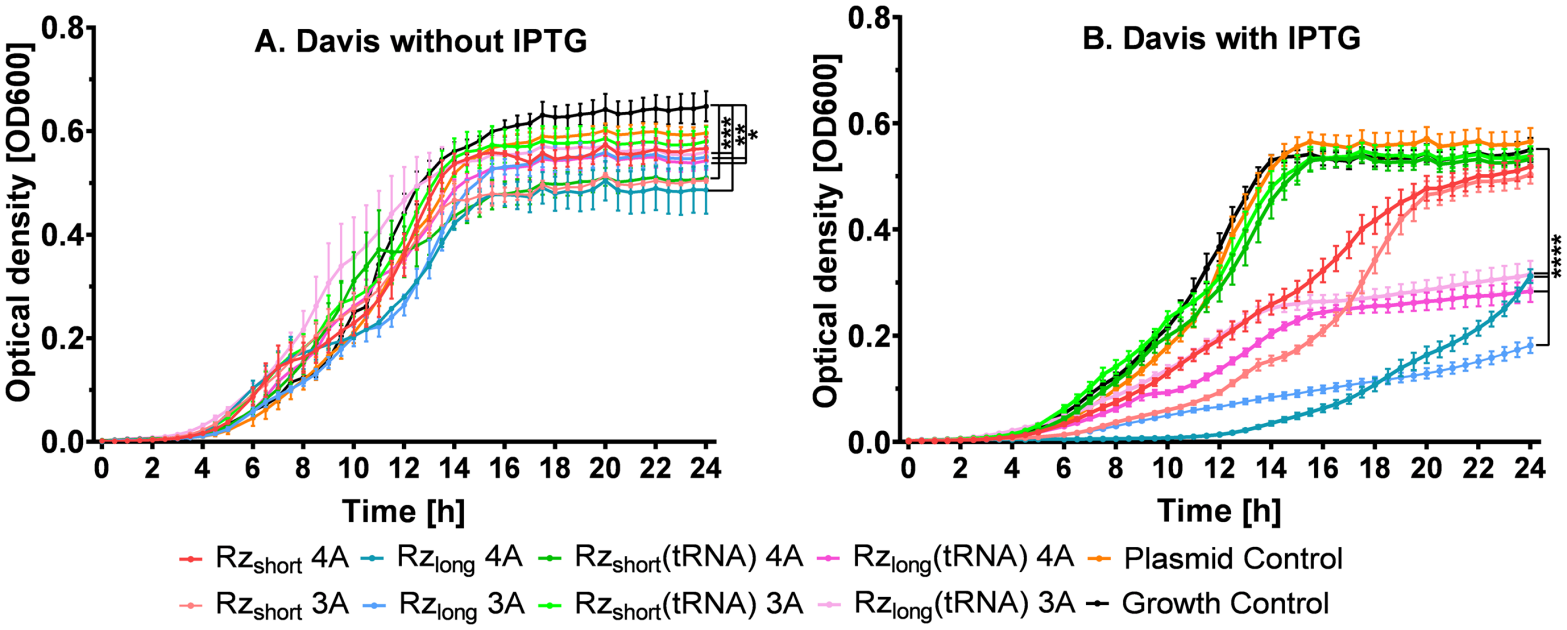
Growth kinetics of *E. coli* BL21(DE3) (on microplates) in Davis medium without IPTG **(A)** or with IPTG added at the beginning of the experiment **(B)**. Data are presented as mean ± SEM (*n* = 4 for A, *n* = 10 for **B**). Tested ribozymes contained either a three-adenine (3A) or a modified four-adenine (4A) catalytic core. Differences in growth at 24 h between bacteria containing a plasmid (with or without Rz) and Growth Control are statistically significant at ****p < 0.0001, ***p < 0.001, **p < 0.01, and *p < 0.05.

Since the kinetic experiments confirmed that Davis medium provided optimal conditions for observing bacterial growth inhibition in cultures with Rz, we used this medium to test Rz activity in cells, both without and with IPTG induction. We compared modified ribozymes containing an extra adenine in the core (4A) to ribozymes with the original three-adenine (3A) catalytic core (Figure 5). Although statistically significant differences in OD_600_ were observed without IPTG induction (Figure 5A), as expected, adding IPTG significantly accentuated the differences between the Rz variants (Figure 5B).

Figure 5B shows that Rz_short_(tRNA)__4A_ and Rz_short_(tRNA)__3A_ (green lines) were both inactive, exhibiting similar growth patterns to the Growth and Plasmid controls. Their standalone variants, Rz_short_3A_ and Rz_short_4A_, were moderately active (red lines) with the 3A variant (light red line) demonstrating stronger inhibitory activity than its 4A counterpart, particularly between 9 and 18 h; however, after 20 h, their activity was similar. The Rz_long_(tRNA) 3A and 4A variants (pink lines) demonstrated stronger and similar inhibitory effects, with slight differences only between 10 and 15 h, and the final OD after 24 h decreased by approximately 50% compared to the Growth Control (Supplementary Table S4).

Importantly, both Rz_long_ variants (blue lines, Figure 5B) effectively suppressed bacterial growth, exhibiting the largest inhibitory effects. The Rz_long_3A_ construct reduced final OD_600_ values by approximately 70% compared to the Growth Control, representing the most potent inhibitory effect among all tested constructs. The differential effects of tRNA fusion on Rz_short_ versus Rz_long_ variants suggest that structural context influences activity, with the tRNA fusion abolishing activity in Rz_short_ constructs while preserving it in Rz_long_ constructs.

Since the kinetic experiments shown in Figure 5 indicated that Rz_long_ and Rz_long_(tRNA) were the most active constructs, we redesigned these ribozymes into inactive variants (Supplementary Figure S6) to determine whether the observed inhibition arises from mRNA blocking or cleavage (Supplementary Figure S16). Interestingly, the growth inhibition of bacterial cells containing the inactive ribozymes (Supplementary Figure S16) was greater than the inhibition caused by their corresponding active versions (Figure 5).

Notably, all active ribozyme variants not only reduced final OD values but also delayed the onset of the exponential growth phase, with Rz_long_ variants showing delays of approximately 4–6 h compared to controls. For Rz_long_, the variant with four adenines (4A) inhibited bacterial growth until ~12 h while the variant with three adenines (3A) did so for ~7 h (Figure 5B). Although their inhibitory effects were similar at 18.5 h, thereafter Rz_long_3A_ showed greater growth inhibition. At 24 h, bacterial growth relative to the Growth Control was 57% for Rz_long_4A_ and 33% for Rz_long_3A_ (Supplementary Table S4), suggesting that the original catalytic core design maintains optimal activity *in vivo*. This statistically significant ~24% difference in final OD between the cultures containing Rz_long_4A_ and Rz_long_3A_ suggests the involvement of mRNA cleavage, as the 3A variant is more catalytically active *in vitro*. If growth inhibition were the result of only translational blocking, the inhibitory activities for Rz_long_3A_ and Rz_long_4A_ should be the same, as they share identical binding arms.

### 3.6 Exploring the potential of inhibition of *Escherichia coli* growth by the ribozyme and tetracycline

**Figure 6:**
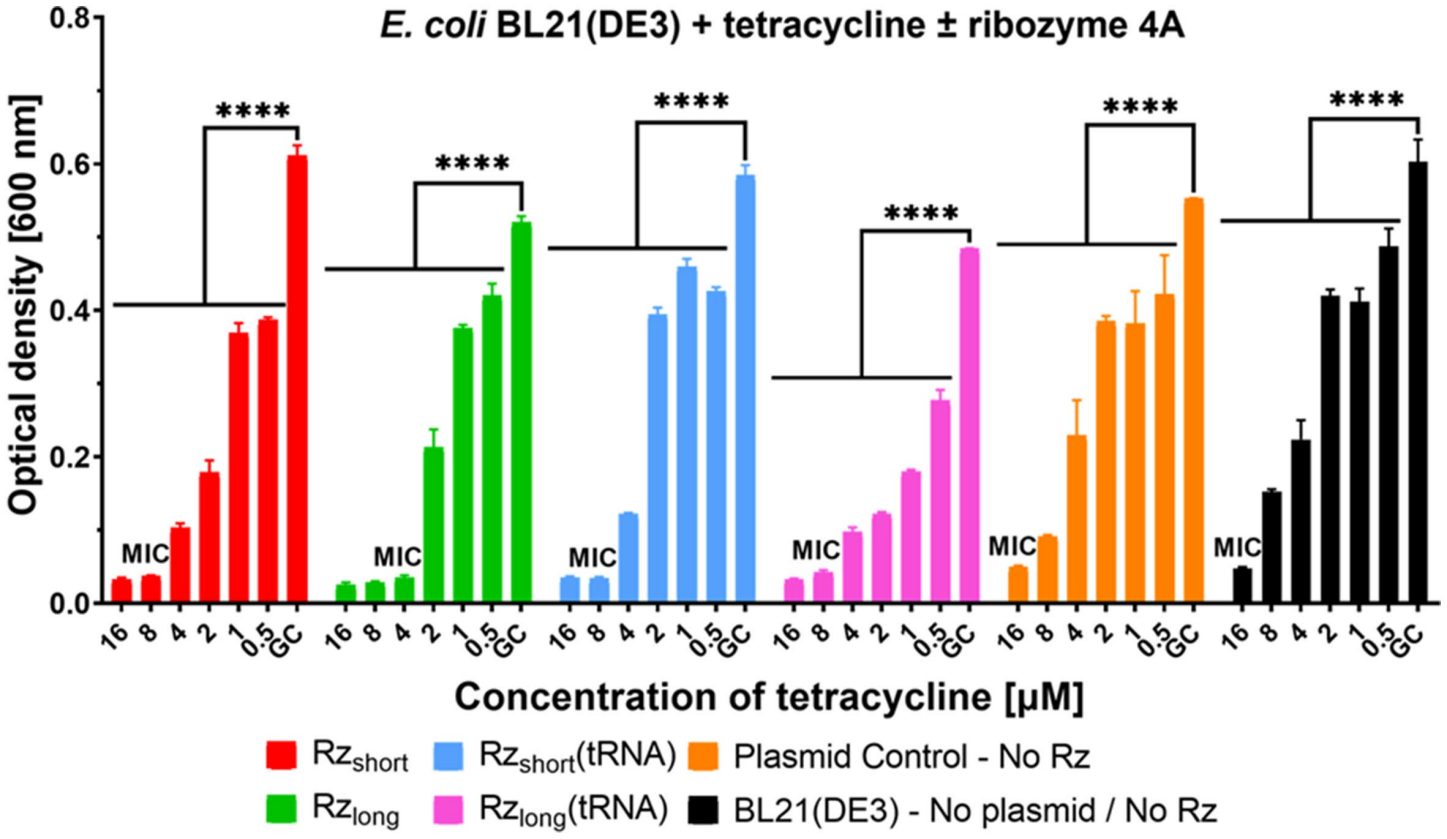
Growth inhibition of *E. coli* BL21(DE3) by ribozymes and tetracycline. Optical density measured after 20 h for *E. coli* BL21(DE3) containing Rz 4A variants or controls, cultured in MHB medium with varying concentrations of tetracycline. Each color represents a different construct as indicated in the legend. Data are presented as mean ± SEM, *n* = 2–5. Differences in optical density relative to Growth Control (GC) are statistically significant (****p < 0.0001). MIC denotes Minimum Inhibitory Concentration, the lowest tetracycline concentration that prevents visible bacterial growth.

To investigate whether ribozyme activity in cells could enhance antibiotic efficacy, we performed a preliminary study using tetracycline. We chose tetracycline due to its relatively high MIC of 16 μM (Wojciechowska et al., 2020) against the *E. coli* BL21(DE3) strain. This experiment was carried out in MHB because it is a commonly used medium for MIC testing. Analysis of the OD of bacterial cultures incubated with tetracycline showed that the presence of Rz variants reduced the MIC of tetracycline to 8 μM for Rz_short_, Rz_short_(tRNA), Rz_long_(tRNA) (both the 4A and 3A variants), and Rz_long_3A_, and to 4 μM for the Rz_long_4A_ variant (Figure 6; Supplementary Figure S17). Consequently, bacteria expressing Rz variants were more susceptible to tetracycline. This synergistic effect represents a twofold reduction in MIC for most ribozyme variants and a fourfold reduction for Rz_long_4A_. The Plasmid Control showed no change in tetracycline MIC compared to the Growth Control, confirming that the enhanced tetracycline susceptibility results from ribozyme activity rather than plasmid burden.

The growth kinetics of cultures incubated with tetracycline and Rz 4A ribozyme variants are shown in Supplementary Figure S18. At increasing tetracycline concentrations, Rz_long_(tRNA) most strongly suppressed the logarithmic growth phase compared to the other Rz 4A variants. However, the standalone Rz_long_ variant caused the greatest reduction in the tetracycline MIC. The growth kinetics of cultures incubated with tetracycline and the Rz 3A variants (Supplementary Figure S19) showed a similar trend to the 4A ribozymes, but with slightly less growth inhibition.

## 4 Discussion

In this study, we designed different versions of ribozymes directed at the *E. coli acpP* mRNA transcript encoding the essential protein ACP. A fragment of the mRNA covering the start codon served as a target substrate for hybridization and potential cleavage by the ribozymes. Using ITC, we confirmed the formation of tight mRNA substrate-ribozyme complexes *in vitro*, with K_a_ values of 10^7^ M^−1^ for Rz_short_ and 10^8^ M^−1^ for Rz_long_ (Table 2). Using gel electrophoresis, we subsequently verified the *in vitro* cleavage of the mRNA fragment by the short-armed (Figure 4) and long-armed ribozymes (Supplementary Figure S1). By incorporating the Rz variants into plasmids and transforming them into *E. coli* BL21(DE3) cells, we evaluated the efficiency of growth inhibition by the intracellularly expressed Rz variants (Figure 5). We also observed that bacteria expressing Rz were more susceptible to tetracycline (Figure 6).

*In vitro*, the cleavage products from the mRNA fragment appeared faster and the gel bands were more intense for Rz_short_ (Figure 4). We attribute this to the formation of a tighter complex between Rz_long_ and the mRNA target (Table 2), which could limit the rate of product release and thus slow catalytic turnover (Supplementary Figure S1). In contrast, *in vivo*, we found that Rz_long_ and Rz_long_(tRNA) were the most effective in inhibiting bacterial growth (Figure 5).

However, the observed *in vivo* inhibition could be attributed to several factors: (i) cleavage of the mRNA by Rz (an effect supported by comparing *in vivo* activity of the 3A and 4A variants); (ii) steric blocking of the mRNA fragment by Rz, which even without cleavage, could prevent translation and consequently ACP synthesis (an effect confirmed using catalytically inactive variants); (iii) cellular RNase H-mediated cleavage of the mRNA: Rz duplex (Jarvis et al., 1996); and (iv) reduced bacterial growth due to plasmid burden, potentially leading to growth inhibition independent of ACP translation disruption. However, we confirmed that this latter contribution was minimal in Davis medium, as we observed only a slight difference between the Growth Control and Plasmid Control samples (Figure 5; Supplementary Tables S3, S4). These effects may also co-exist, with each contributing to the observed inhibition of bacterial growth.

Nevertheless, the expression of Rz_long_ resulted in the most substantial inhibition of bacterial growth (Figure 5). For bacteria expressing Rz_short_, growth was also inhibited, but rebounded earlier. This could be attributed to faster degradation of Rz_short_ by endogenous ribonucleases, preventing it from reaching the substrate swiftly. An alternative explanation could be weaker binding of Rz_short_ to the mRNA target *in vivo*, contrary to the *in vitro* ITC data. Another observation is that embedding Rz in the tRNA structure does not necessarily improve its efficiency, although it still results in noticeable growth inhibition, particularly by Rz_long_(tRNA), which was slightly more effective for the 4A variant (Figure 5). Possibly, it is more difficult for Rz to access and bind to the target when it is embedded within the tRNA, regardless of the additional linker (Supplementary Figure S4). Conversely, the tRNA structure might protect Rz from degradation. However, the intracellular lifetime of the Rz variants in bacterial cells was not assessed. Moreover, sequences adjacent to the target sequence in bacterial *acpP* mRNA also form three-dimensional conformations, which may hinder the ribozyme from effectively binding to the mRNA substrate. Further studies using a longer fragment of the mRNA substrate could clarify the differences between the in vitro and *in vivo* activities of the tested ribozyme versions.

We also tested different media for bacterial cultures (Supplementary Figures S8–S15). Notably, the doubling time of *E. coli* is approximately 20 min in a nutrient-rich medium under aerobic laboratory conditions (Gibson et al., 2018), whereas in the human gut, an anaerobic environment, the doubling time extends to ~40 h (Savageau, 1983). Therefore, even when using minimal media, laboratory conditions are still more favorable for bacterial growth compared to those in the human gut.

When comparing the activity of ribozymes with three or four adenines in the catalytic core, the differences are not entirely clear, revealing complex, time-dependent effects. After 24 h of culture, both variants (4A and 3A) of Rz_short_ showed similar antibacterial activity (Figure 5). However, at earlier time points, the 3A variant showed a stronger effect. For the Rz_long_ ribozyme, the 4A version inhibited bacterial growth for 5 h longer than the 3A variant (Figure 5). However, after 24 h, the 3A variant showed a 24% greater reduction in final growth than the 4A variant (Figure 5). This time-dependent pattern suggests different kinetic properties of the variants, with the 4A construct potentially having a more rapid initial activity but lower long-term stability or efficiency compared to the 3A variant. For Rz_long_(tRNA), the 4A variant inhibited bacterial growth slightly more effectively at early time points, but from the 15^th^ hour of culture, the level of inhibition became similar (Figure 5). Although these differences are not entirely clear, our observations suggest that modifying the catalytic core with additional adenines may be important for optimizing cleavage of bacterial mRNA. It is promising that the long-armed variants of the 4A ribozyme inhibit bacterial growth more effectively at early time points than the 3A variants.

The presence of Rz in bacteria increases their sensitivity to tetracycline, indicating an additive or even synergistic effect (Figure 6). Synergy between antibacterial compounds has been studied since the early 1950s (Acar, 2000), but typically antibiotic/antibiotic (Karki et al., 2021; Malki et al., 2023) or antibiotic/peptide (Macyszyn et al., 2023; Zhou and Peng, 2013) combinations have been investigated. However, synergies between antibiotics and antisense oligonucleotides can also be valuable. For example, we previously observed synergistic effects between antibiotics and 10-mer PNA oligomers targeting the essential *acpP* mRNA, the same target as in this study (Castillo et al., 2018). Combinations of PNA_anti-*acpP*_ with polymyxin B and trimethoprim were synergistic in *E. coli* strains (Castillo et al., 2018) and reduced the MIC four- to eightfold for PNA and four- to 13-fold for the antibiotics, depending on the combination. We have also shown synergy between PNA_anti-*thyA*_ targeting the non-essential *thyA* gene, encoding an enzyme involved in folic acid metabolism, and trimethoprim in *E. coli* (Równicki et al., 2018). The combinations showed a fourfold lower MIC for PNA and a 16-fold lower MIC for the antibiotic. Therefore, it was interesting to observe in this study that the expression and activity of Rz made bacteria more susceptible to tetracycline, especially for the Rz_long_4A_ variant (Figure 6). This enhanced tetracycline susceptibility may result from the combined stress of a ribosome-targeting antibiotic and ribozyme-mediated disruption of an essential transcript. Particularly worthy of further investigation into potential synergistic effects with ribozymes are β-lactam antibiotics, as they are the most commonly used antibiotics. β-lactams have been studied for synergistic effects with other antibiotics (Gutmann et al., 1986; Story-Roller et al., 2019) or peptides (Zhang et al., 2021), showing their potential to interact with other compounds to inhibit bacterial growth. To the best of our knowledge, β-lactams have not been studied for synergies with ribozymes, so it would be worth exploring such combinations.

Although targeted cleavage of bacterial mRNA seems a compelling research area, this approach has not been extensively investigated. The essential bacterial *acpP* gene was previously not tested as an RNA enzyme target. The few previous studies applying a similar approach used different targets. Only one study (Tan et al., 2004) targeted an essential bacterial gene, *ftsZ*, but used a DNA enzyme, so a direct comparison with our approach is not possible. In bacteria, the hammerhead ribozyme-mediated mRNA cleavage was previously only observed on the mRNA of non-essential *lexA* and *lacZ* genes (Junn and Kang, 1996; Yadava et al., 2005), but the assays were designed to report a phenotype change (change of color of the culture or inducing cell sensitivity to UV light) and not to cause growth inhibition. In one case, ribozyme *cis*-cleavage activity was reported to block the overexpression of the toxin encoded by the *ibsC* gene (Huang et al., 2019). Other substrates for cleavage included a proto-oncogene, but this mRNA was encoded on a plasmid and the cleavage was mediated by a larger enzyme—the RNase P ribozyme (Toumpeki et al., 2018). Therefore, the inhibition of bacterial growth observed in our study for 14 h by targeting an essential gene expands the potential of ribozyme applications in bacterial cells. In another study, the delta ribozyme showed high efficiency against the industrially important Gram-positive bacterium *L. lactis*, but its active structure is more complex (Fiola et al., 2006) in contrast to the simpler hammerhead Rz 4A and 3A variants used in our study.

Certainly, key considerations in such studies include selecting an appropriate mRNA substrate and an enzyme capable of cleaving that target effectively to prevent the bacteria from recovering. Future research should focus on developing more effective, non-invasive methods for ribozyme delivery into cells, moving beyond reliance on plasmids and specialized bacterial strains that provide T7 polymerase. While plasmids are useful for initial research, designing and preparing a suitable expression cassette (and the plasmid itself) is complex and time-consuming. This complexity limits the practical application of catalytic enzymes in cells. For ribozymes, in particular, finding effective methods for protection against nuclease degradation is crucial.

Nevertheless, ribozymes have the potential for use as antibacterial agents or to enhance existing antibiotics, potentially allowing for lower effective concentrations. Our sequence alignment shows that the targeted mRNA region encoding ACP is 100% conserved among the corresponding fragments in many *E. coli* pathogenic strains, indicating that this transcript is a potent target for ribozyme cleavage in these clinically relevant strains. We confirmed that an RNA enzyme has the ability to cleave the mRNA of this essential gene and consequently lead to inhibition of bacterial growth, not merely cause a change in phenotype. Furthermore, we observed growth inhibition for up to 24 h, which is longer than previously reported, showing promise for sustained activity. We also showed that adding an extra adenine in the catalytic core does not preclude inhibition of bacterial growth, although *in vitro* the 3A variant was more effective. Overall, we believe that using a small ribozyme is an advantage compared to other larger RNA or DNA enzymes.

Our findings, alongside existing literature, demonstrate that targeting essential bacterial mRNA with specifically designed ribozymes warrants further research. Theoretically, a ribozyme can be tailored to selectively target any designated bacterial mRNA in all bacterial strains where the DNA sequences of essential proteins are known. A ribozyme can also be engineered to target different mRNA sequences within a single strain. If pathogenic strains share the same substrate sequence, a single specific ribozyme could effectively target all such strains. Additional nucleotides in the catalytic core can improve the antibacterial catalytic activity of ribozymes. Furthermore, the combination of a ribozyme with an antibiotic presents an opportunity to enhance the antibiotic’s efficacy while potentially reducing its required bactericidal concentration. Importantly, the bacterial target studied in this work differs from the human ACP (Park et al., 2019). In the first step, it was important to determine whether we could inhibit bacterial growth by targeting the *acpP* mRNA. However, future studies should verify whether the homologous human mRNA contains a similar sequence to assess the risk of non-specific binding of the ribozyme to human mRNAs.

## Data Availability

The original contributions presented in the study are included in the article/Supplementary material, further inquiries can be directed to the corresponding author.
