# Supplementary material for: Hammerhead ribozymes directed against mRNA of an essential gene inhibit Escherichia coli growth and enhance tetracycline efficacy
Source: Front Microbiol. 2025 Oct 3;16:1663476. doi: 10.3389/fmicb.2025.1663476 (PMC12531169; doi:10.3389/fmicb.2025.1663476)
Supplement: Supplementary file 1 [file Data_Sheet_1.pdf]

Supplementary Material

for

Hammerhead ribozymes directed against mRNA of an essential gene inhibit *Escherichia coli* growth and enhance tetracycline efficacy

Joanna Miszkiewicz-Golec[1], Ksenia Maximowa[1], Maciej Łukaszewicz[2], Dariusz Bartosik[3], Edward Darżynkiewicz[1,2,†] and Joanna Trylska[1*]

[1]University of Warsaw, Centre of New Technologies, Warsaw, Poland

[2]University of Warsaw, Faculty of Physics, Institute of Experimental Physics, Division of Biophysics, Warsaw, Poland

[3]University of Warsaw, Faculty of Biology, Institute of Microbiology, Department of Bacterial Genetics, Warsaw, Poland

[†]Deceased

*correspondence: joanna@cent.uw.edu.pl

Supplementary Table S1. Alignment of the DNA sequences flanking the start codon of the *acpP* cleavage site *ATG* in selected pathogenic *E. coli* strains compared to the reference non-pathogenic *E. coli* BL21(DE3) strain. Genomic sequences were retrieved from the Kyoto Encyclopedia of Genes and Genomes (KEGG) for Bacteria Organisms database (Kanehisa 2000), aligned using the Microbial Nucleotide BLAST blastn program (Altschul 1997), and compared in the FASTA format in BioEdit Sequence Alignment Editor (Hall 1999).

Pathotype group	*Escherichia coli* strain	Sequence ID:Range	% of DNA alignment
non-pathogenic	BL21(DE3) (Jeong et al. 2009)	NC_012971.2:1154164-1154197	GATAGGAAATTTAAGAGT*ATG*AGCACTATCGAAG
EAEC	O44:H18 042 (Chaudhuri et al. 2010)	NZ_CP042934.2:1178363-1178396	100%
EHEC	O157:H7 EDL933 (Perna et al. 2001)	NZ_CP008957.1:1598427-1598460	100%
EHEC	O157:H7 Sakai (Makino 1998)	NC_002695.2:1510833-1510866	100%
EHEC	O145:H28 RM13514 (Cooper et al. 2014)	NZ_CP006027.1:1377026-1377059	100%
EPEC	O127:H6 E2348/69 (Iguchi et al. 2009)	NC_011601.1:1235926-1235959	100%
EPEC	O55:H7 RM12579 (Kyle et al. 2012)	NC_017656.1:1402712-1402745	100%
ETEC	O139:H28 E24377A (Rasko et al. 2008)	NC_009801.1:1229028-1229061	100%
ETEC	O78:H11:K80 H10407 (Crossman et al. 2010)	NC_017633.1:1257657-1257690	100%
ETEC	UMNK88 (Shepard et al. 2012)	NC_017641.1:1332353-1332386	100%
UPEC	O6:K2:H1 CFT073 (Welch et al. 2002)	NZ_CP051263.1:1303345-1303378	100%
UPEC	O18:K1:H7 UTI89 (Chen et al. 2006)	NZ_CP064825.1:1196561-1196594	100%
UPEC-ESBL	O25b:K100:H4-ST131 EC958 (Forde et al. 2014)	NZ_HG941718.1:1211962-1211995	100%

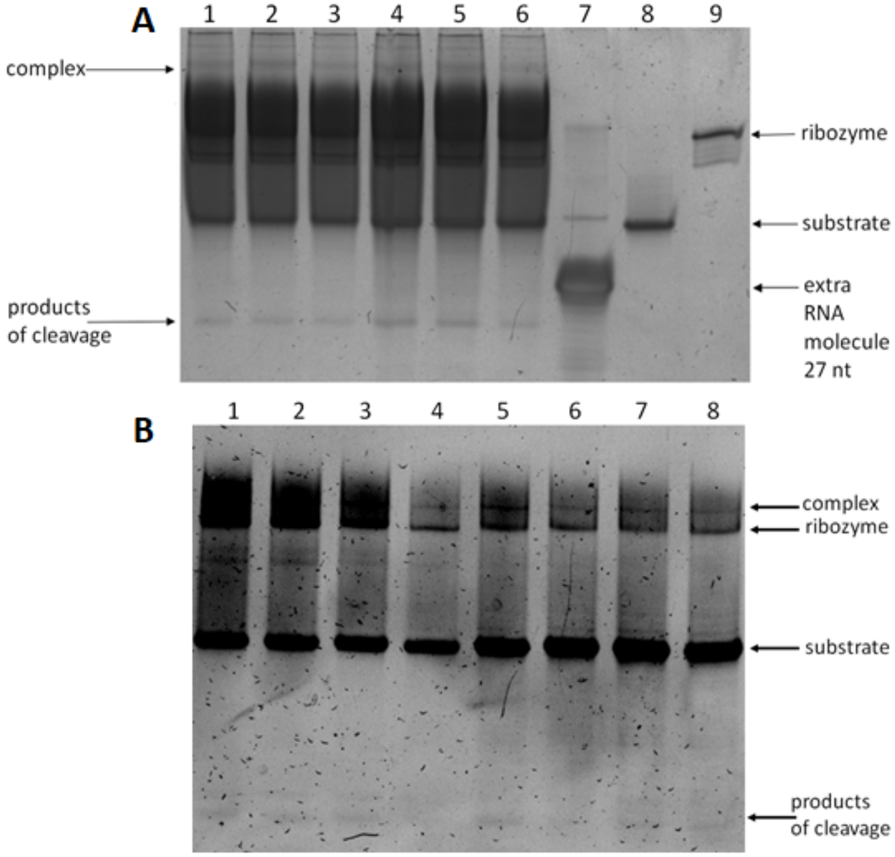

Supplementary Figure S1. *In vitro* cleavage of the mRNA$_{acpP}$ substrate by Rz$_{long_4A}$ (denoted ribozyme in the figure). **(A)** A 20% denaturing polyacrylamide gel (with 8 M urea) showing cleavage at different temperatures and incubation times: lane 1 – 30 minutes at 25°C; lane 2 – 30 minutes at 37°C; lane 3 – 15 minutes at 55°C; lane 4 – 10 minutes at 70°C; lane 5 – 4 minutes at 80°C; lane 6 – 3 minutes at 90°C. Controls: lane 7 – an extra RNA molecule (27 nt) used as a marker for size comparison of the cleavage products (18 and 20 nt); lane 8 – mRNA$_{acpP}$ substrate alone; lane 9 – Rz$_{long_4A}$ alone. Reactions were stopped with the 75 mM EDTA. Samples 1 – 6 were loaded into wells immediately after incubation. For samples incubated below 70°C, they were additionally heated at 70°C for 3 min before electrophoresis. **(B)** An 18% denaturing polyacrylamide gel (with 7 M urea) showing substrate cleavage by Rz$_{long_4A}$ after 6-hour incubation at different Rz$_{long_4A}$:substrate molar ratios: lane 1 – 2.5:1; lane 2 – 2:1; lane 3 – 1.5:1; lane 4 – 1:1; lane 5 – 1:1.5; lane 6 – 1:2; lane 7 – 1:2.5; lane 8 – 1:3.

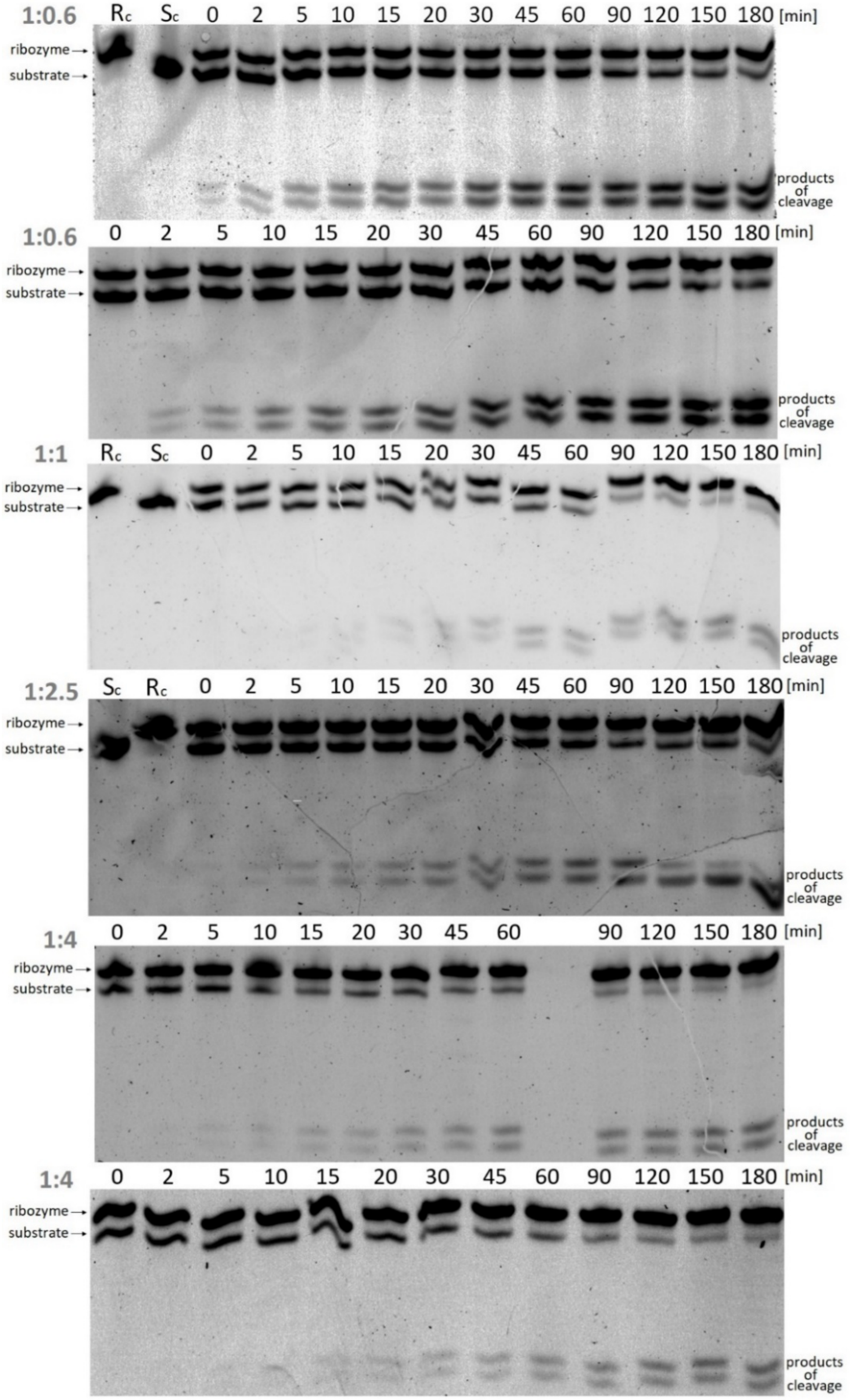

Supplementary Figure S2. *In vitro* cleavage of the mRNA substrate by Rz_{short_4A} (denoted ribozyme) over time (0 – 180 min) at different substrate:ribozyme molar ratios (indicated in the top left corner of each gel). 15% denaturing polyacrylamide gels with 7 M urea show cleavage kinetics. Where shown, control samples include S_C – substrate alone; R_C – ribozyme alone.

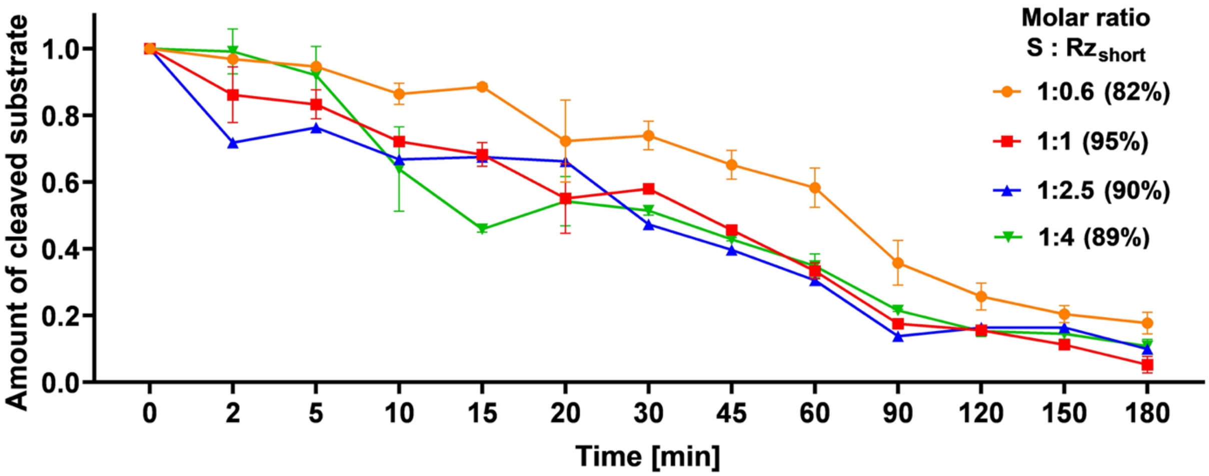

Supplementary Figure S3. *In vitro* cleavage of the mRNA$_{acpP}$ substrate by Rz$_{short_4A}$ at different molar ratios after 3-hour incubation. Data are normalized and presented as the mean ± SEM (n = 2 for 1:0.6, 1:1, and 1:4 ratios; n = 1 for 1:2.5 ratio). Values in parenthesis indicate the percentage of substrate cleaved after 180 min of incubation.

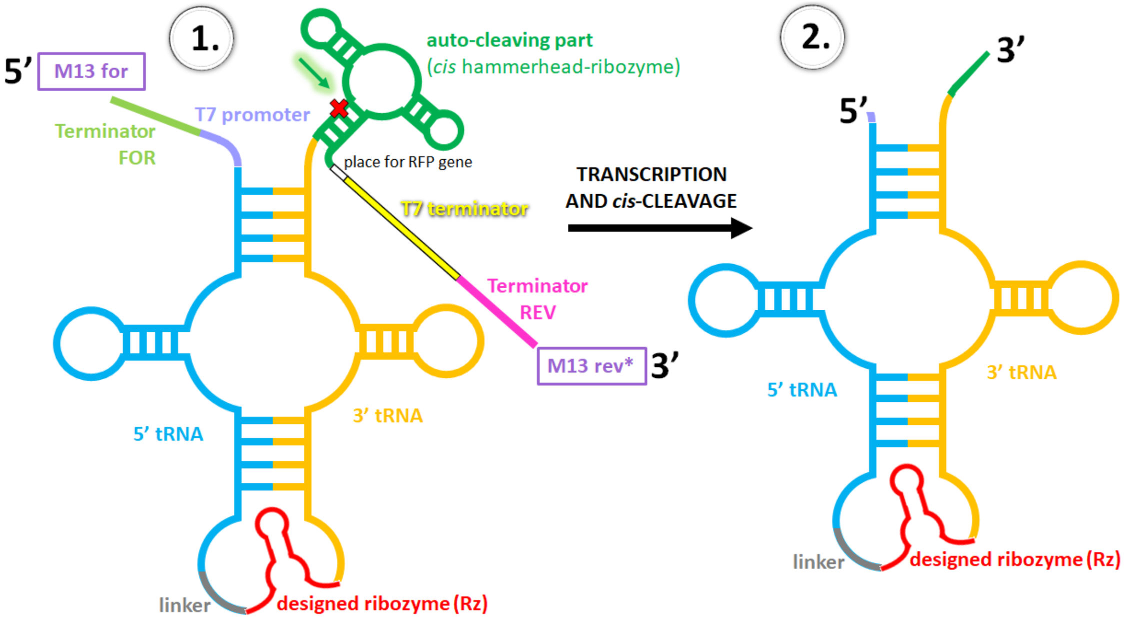

Supplementary Figure S4. 1. Schematic representation of the cassette with Rz(tRNA) encoded on the pUC57-Kan plasmid. Fragment colors correspond to sequence coloring in Table S2. Restriction enzyme sites are not shown. The green arrow and red cross indicate the cleavage site of the auto-cleaving part. **2.** Predicted position of the Rz structure within the tRNA scaffold after transcription from the plasmid and self-cleavage of the *cis*-cleaving ribozyme.

Supplementary Table S2. General organization and sequences of the ribozyme expression cassettes cloned into pUC57-Kan. Terminator_rev* and M13_rev* are encoded on a complementary strand in the 5' to 3'direction. Entries without background coloring indicate restriction enzymes. CCCCCCAAAAAA – represents an extra sequence between restriction enzymes. The **A** letter in bold indicates the extra adenine in the 4A-ribozyme variants.

General scheme of the cassette with ribozyme – leading strand 5' → 3'

FspI – M13_for – SphI – Terminator_ for – T7 promoter – 5'tRNA+linker – Hammerhead ribozyme – 3'tRNA – auto-cleaving part – EcoRI – CCCCCCAAAAAA– SpeI – T7 terminator– Terminator_rev* – AgeI – M13_rev* – PciI

Terminator_for (ilvBN natural terminator)   Terminator_rev* (ECK120016882 natural terminator)

The sequence of the cassette with Rz$_{short}$ – leading strand 5' → 3'

TGCGCAGTAAAACGACGGCCAGTGCATGCAAGACCCCCGCACCGAAAGGTCCGGGGGTTTTTTTTAAT
ACGACTCACTATAGGTGCTCACTGATGAGGCCGAAAGGCCG**A**AAACTCTTAAAGTCCTCTACGAAAGTA
GAGCTGATGAGAGCGAAAGCTCGAAACGAATTCCCCCCCAAAAAAACTAGTCTAGCATAACCCCTTGGG
GCCTCTAAACGGGTCTTGAGGGGTTTTTTGCATAAAAAAACCCGCTTGCGCGGGCTTTTTCACAACCGGT
GTCATAGCTGTTTCCTGACATGT

The sequence of the cassette with Rz$_{long}$ – leading strand 5' → 3'

TGCGCAGTAAAACGACGGCCAGTGCATGCAAGACCCCCGCACCGAAAGGTCCGGGGGTTTTTTTTAAT
ACGACTCACTATAGGACTTCGATAGTGCTCACTGATGAGGCCGAAAGGCCG**A**AAACTCTTAAATTTCCTA
TCGGTCCTCTACGAAAGTAGAGCTGATGAGAGCGAAAGCTCGAAACGAATTCCCCCCCAAAAAAACTAG
TCTAGCATAACCCCTTGGGGCCTCTAAACGGGTCTTGAGGGGTTTTTTGCATAAAAAAACCCGCTTGCGC
GGGCTTTTTCACAACCGGTGTCATAGCTGTTTCCTGACATGT

The sequence of the cassette with Rz$_{short}$(tRNA) – leading strand 5' → 3'

TGCGCAGTAAAACGACGGCCAGTGCATGCAAGACCCCCGCACCGAAAGGTCCGGGGGTTTTTTTTAAT
ACGACTCACTATAGGCCCGGATAGCTCAGTCGGTAGAGCAGCGGCCGTACTTCCACCAACGAGTCCAGT
GCTCACTGATGAGGCCGAAAGGCCG**A**AAACTCTTAAATAATACGGCCGCGGGTCCAGGGTTCAAGTCCC
TGTTCGGGCGCCGTCCTCTACGAAAGTAGAGCTGATGAGAGCGAAAGCTCGAAACGAATTCCCCCCCAA
AAAAACTAGTCTAGCATAACCCCTTGGGGCCTCTAAACGGGTCTTGAGGGGTTTTTTGCATAAAAAAACC
CGCTTGCGCGGGCTTTTTCACAACCGGTGTCATAGCTGTTTCCTGACATGT

The sequence of the cassette with Rz$_{short}$(tRNA) – leading strand 5' → 3'

TGCGCAGTAAAACGACGGCCAGTGCATGCAAGACCCCCGCACCGAAAGGTCCGGGGGTTTTTTTTAAT
ACGACTCACTATAGGCCCGGATAGCTCAGTCGGTAGAGCAGCGGCCGTACTTCCACCAACGAGTCCAGA
CTTCGATAGTGCTCACTGATGAGGCCGAAAGGCCG**A**AAACTCTTAAATTTCCTATCGTAATACGGCCGCG
GGTCCAGGGTTCAAGTCCCTGTTCGGGCGCCGTCCTCTACGAAAGTAGAGCTGATGAGAGCGAAAGCTC
GAAACGAATTCCCCCCCAAAAAAACTAGTCTAGCATAACCCCTTGGGGCCTCTAAACGGGTCTTGAGGG
GTTTTTTGCATAAAAAAACCCGCTTGCGCGGGCTTTTTCACAACCGGTGTCATAGCTGTTTCCTGACATGT

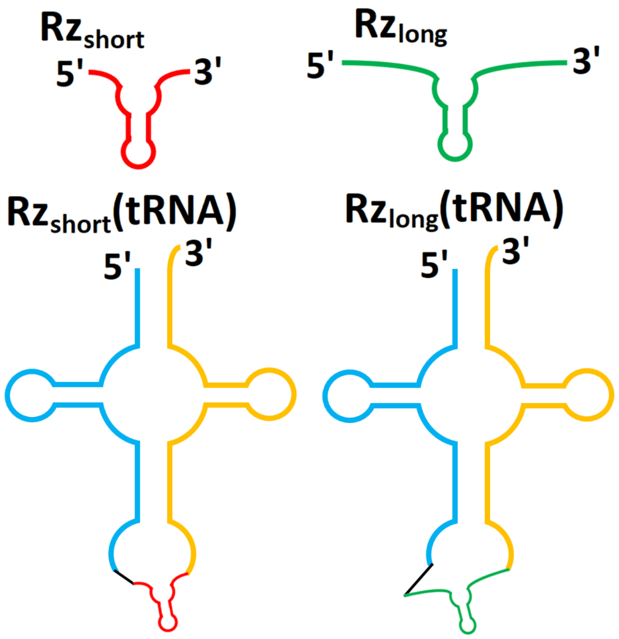

Supplementary Figure S5. Schematic representation of the four ribozyme variants (Rz): standalone and tRNA embedded versions. Color coding: **red** – Rz_{short}; **green** – Rz_{long}; **blue** – 5' tRNA; **dark yellow** – 3' tRNA; **black** – linker).

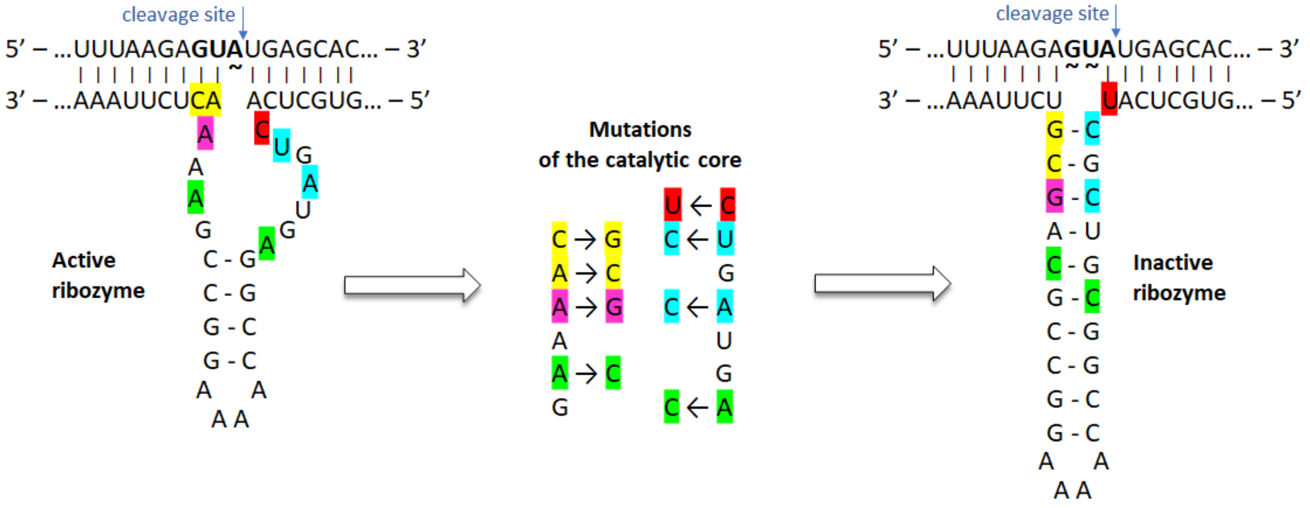

Supplementary Figure S6. Strategy for generating an inactive ribozyme. Nucleotide coloring is random. 5'–…–3' – represents the targeted $mRNA_{acpP}$ substrate fragment with the cleavage site in bold; 3'–…–5' – represents the anti-$mRNA_{acpP}$ ribozyme fragment. Inactive ribozyme variants were obtained using the Q5 Site-Directed Mutagenesis Kit (New England Biolabs) following the manufacturer's protocol and using the following primers:
for Rz_{long}: AAAGGCCGCAGCGTCTTAAATTTCCTATCGGTC (FOR), CGGCCGCAGCGATGAGCACTATCGAAGTC (REV);
for Rz_{long}(tRNA): AAAGGCCGCAGCGTCTTAAATTTCCTATCGTAATACG (FOR), CGGCCGCAGCGATGAGCACTATCGAAGTC (REV).
All mutagenesis reactions used the pUC57 Rz templates.

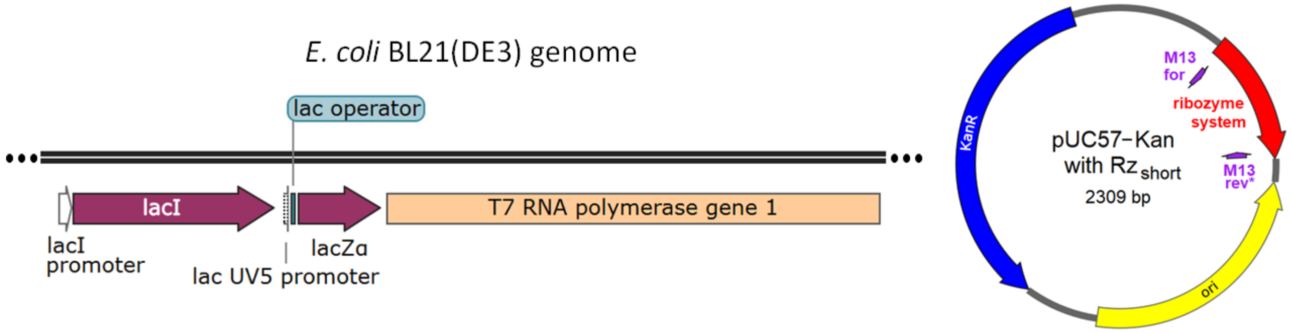

Supplementary Figure S7. *E. coli* BL21(DE3) genome fragment showing the T7 RNA polymerase gene with the lac operon for the IPTG induction, and the plasmid with Rz_{short} transcribed by T7 RNA polymerase. Diagrams were created using SnapGene (www.snapgene.com) based on the *E. coli* BL21(DE3) genome sequence from the KEGG database and plasmid sequence designed in this study.

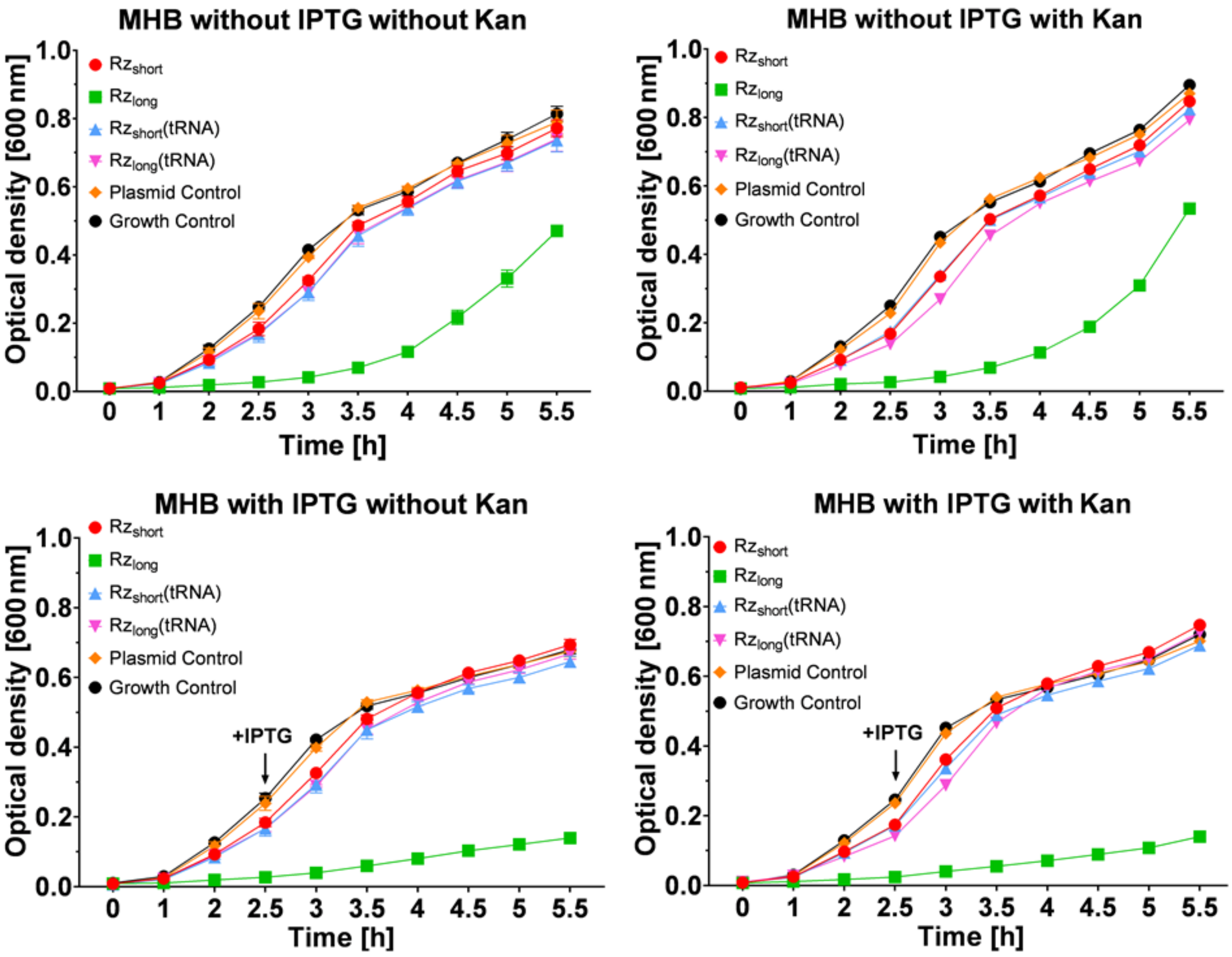

Supplementary Figure S8. *E. coli* BL21(DE3) growth in MHB medium without and with kanamycin (Kan). Cultures were grown in falcon tubes and optical density data are presented as the mean ± SEM (n = 2 for cultures without Kan; n = 1 for cultures with Kan). IPTG was added after 2.5 h of cultivation.

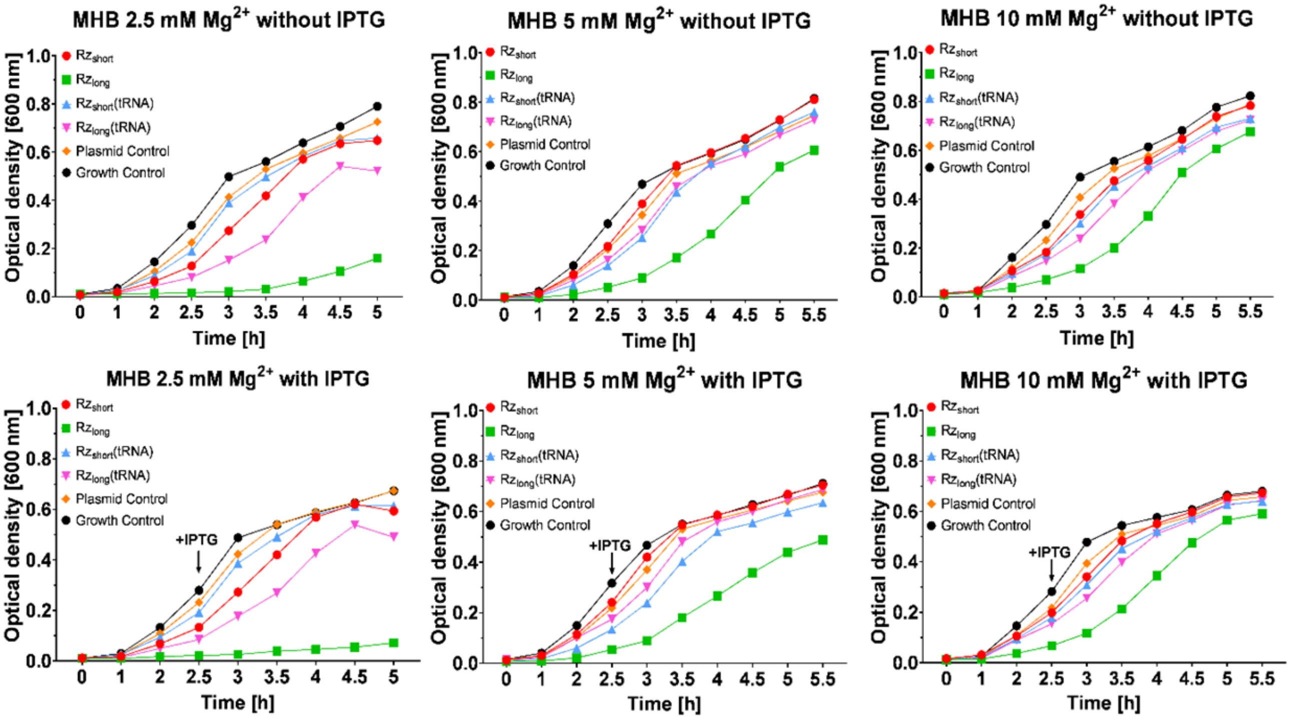

Supplementary Figure S9. Effect of magnesium concentration on *E. coli* BL21(DE3) growth in MHB medium. Cultures were grown in falcon tubes with 2.5, 5, or 10 mM Mg^{2+} (n = 1). IPTG was added after 2.5 h of cultivation.

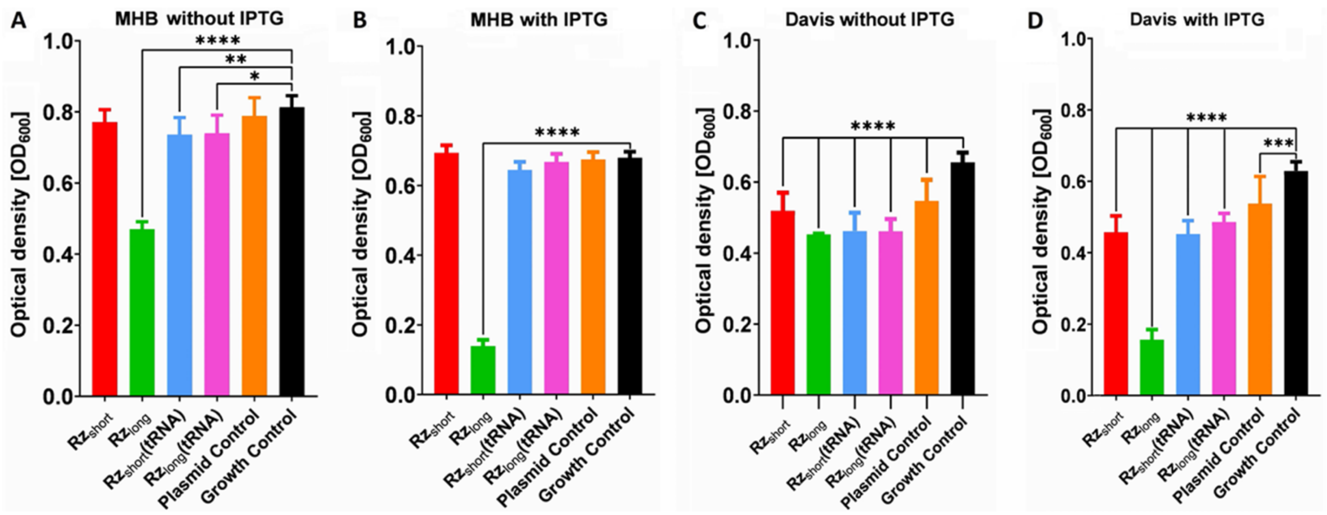

Supplementary Figure S10. Optical density measurements of *E. coli* BL21(DE3) cultures containing different ribozyme-encoding plasmids, grown in falcon tubes and measured at the end of cultivation: 5.5 h in MHB medium (**A**, **B**) and 7 h in Davis medium (**C**, **D**). Panels **A** and **D** show growth with IPTG added after 2.5 h (MHB) or 4 h (Davis) of cultivation. Mean values ± SEM are shown (n = 2). Statistical comparisons are relative to Growth Control and are statistically significant for **** $P < 0.0001$, *** $P < 0.001$, ** $P < 0.01$, * $P < 0.05$; other differences are not significant.

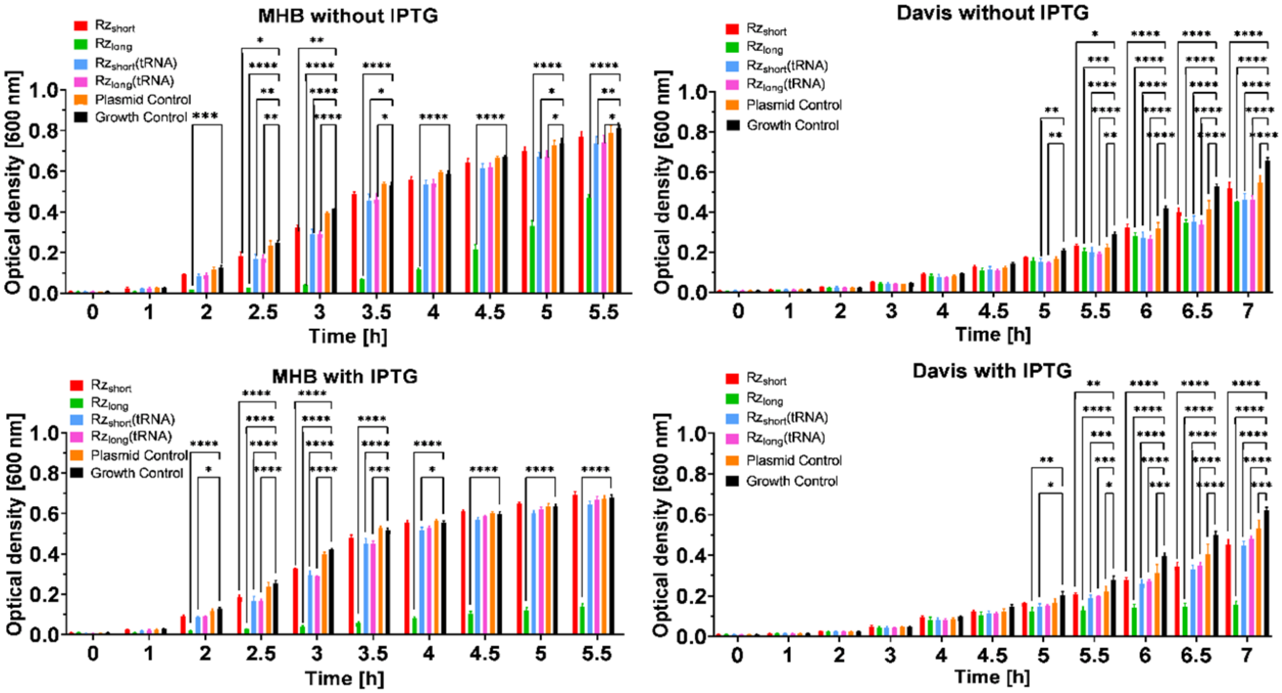

Supplementary Figure S11. Growth kinetics of *E. coli* BL21(DE3) cultures containing ribozyme-encoding plasmids grown in MHB (2 biological replicates) and Davis medium (3 biological replicates), with and without IPTG induction. IPTG was added after 2.5 h in MHB and after 4 h in Davis medium. Mean values ± SEM are shown (n = 6-18 technical replicates). Statistical comparisons are relative to Growth Control and are statistically significant for **** $P < 0.0001$, *** $P < 0.001$, ** $P < 0.01$, and * $P < 0.05$; other differences are not significant.

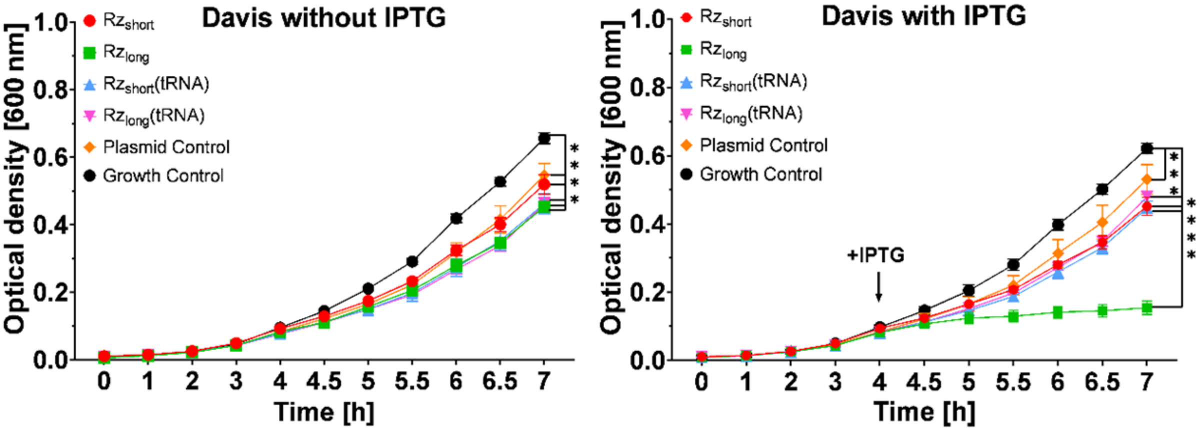

Supplementary Figure S12. Growth kinetics of *E. coli* BL21(DE3) cultures grown in Davis medium in falcon tubes. Mean values ± SEM are shown, n = 2. IPTG was added after 4 h of cultivation. Differences in optical density for plasmid-containing bacteria relative to Growth Control are statistically significant at **** $P < 0.0001$ or *** $P < 0.001$. Comparisons between the same constructs with and without IPTG induction were highly significant at 6, 6.5, and 7 h for Rz_{long} (**** $P < 0.0001$); significant at 5.5 h for Rz_{long} and at 7 h for Rz_{short} (** $P < 0.01$); and moderately significant at 5.5 h for Rz_{short} (* $P < 0.05$), with other time points showing no statistically significant differences.

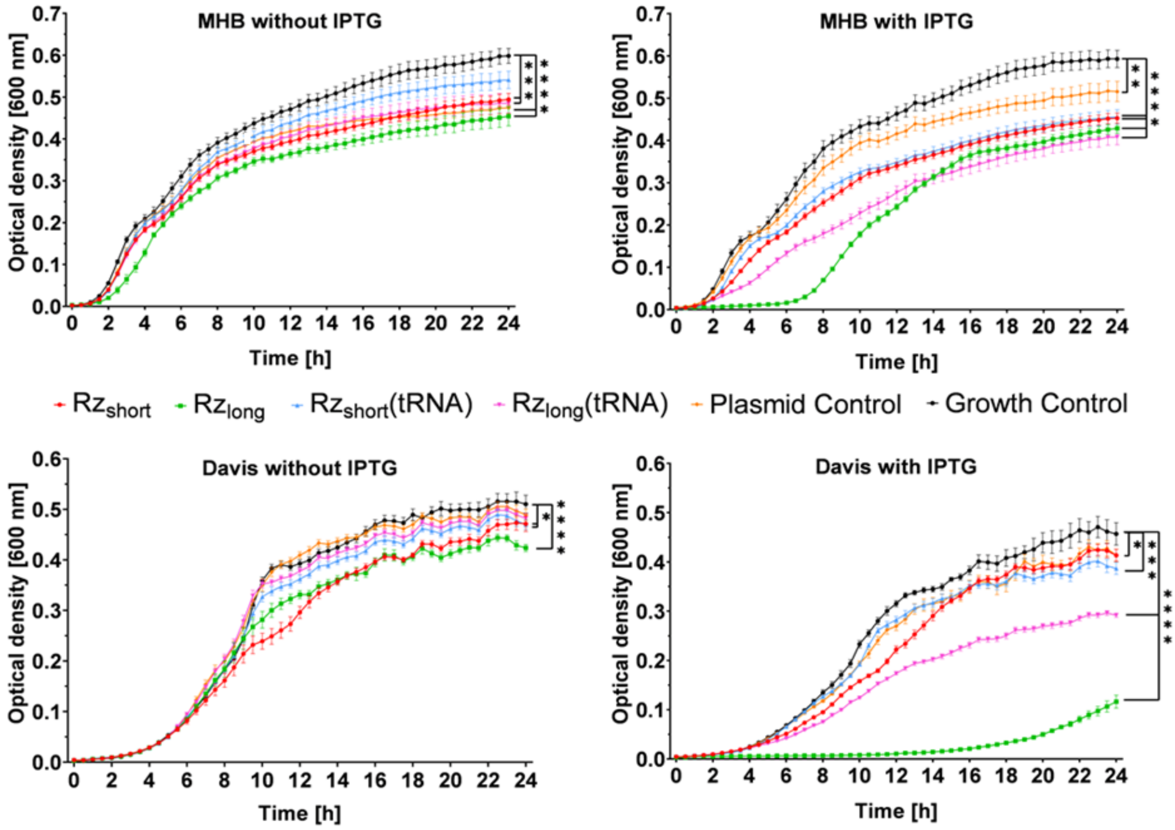

Supplementary Figure S13. Growth kinetics of *E. coli* BL21(DE3) cultures grown on microplates in MHB or Davis medium. IPTG was added at the beginning of the experiments. Mean values ± SEM are shown (n = 6-18 technical replicates). Statistical significance of growth differences between plasmid-containing bacteria and Growth Control is indicated as **** $P < 0.0001$, *** $P < 0.001$, ** $P < 0.01$, * $P < 0.05$, while other differences are not significant.

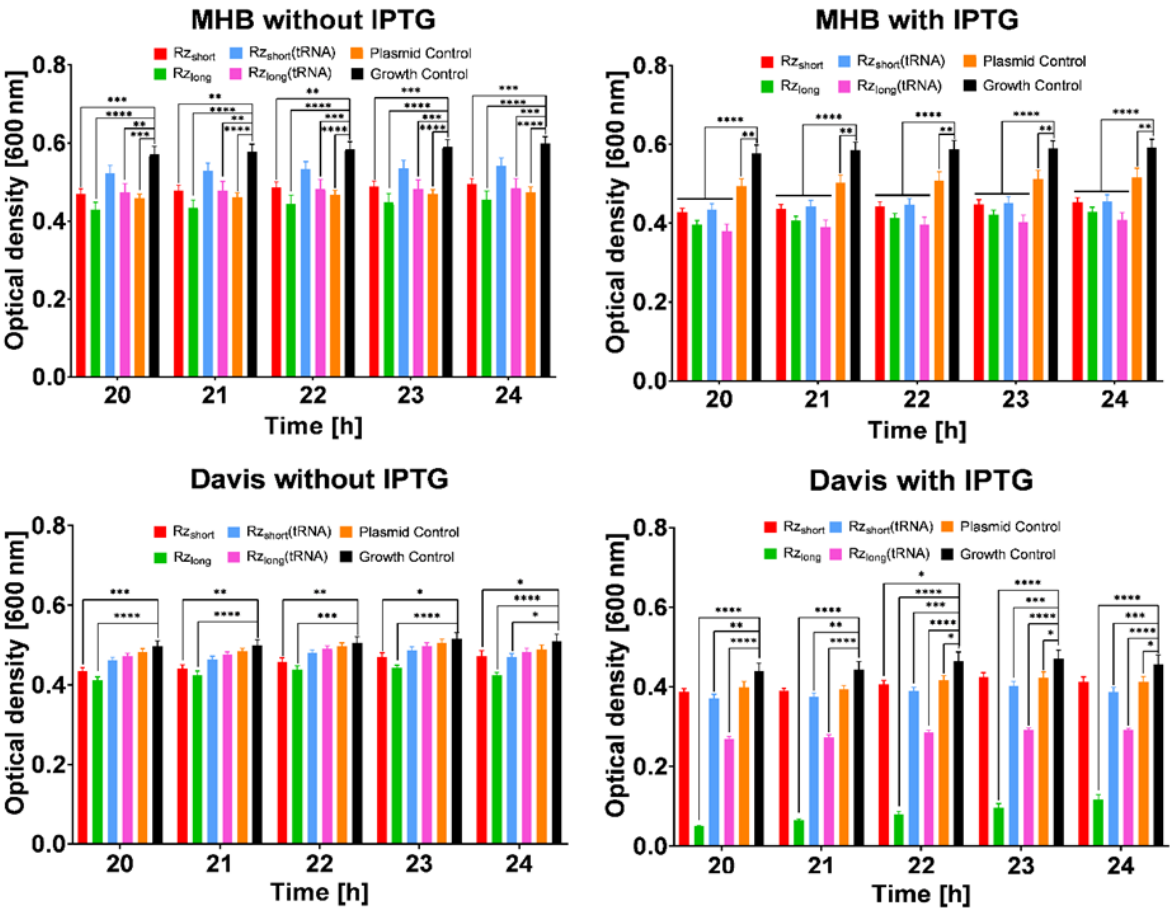

Supplementary Figure S14. Optical density of *E. coli* BL21(DE3) cultures containing different plasmids measured between 20 and 24 h of cultivation. Data are presented as the mean ± SEM (n = 6-18). Statistical comparisons are relative to the Growth Control and are statistically significant at **** P < 0.0001, *** P < 0.001, ** P < 0.01, and * P < 0.05; other differences are not significant.

Supplementary Table S3. *E. coli* BL21(DE3) growth with or without different ribozymes after 24 h of cultivation in MHB or Davis medium. Values represent the percentage of growth relative to the Growth Control, which was set as the 100 % reference.

Conditions	MHB without IPTG [%]	MHB with IPTG [%]	Davis without IPTG [%]	Davis with IPTG [%]
Culture				
Growth Control	100	100	100	100
Plasmid Control	79	87	96	90
Rz_{short}	83	76	92	90
Rz_{long}	76	72	83	**25**
Rz_{short}(tRNA)	90	77	92	85
Rz_{long}(tRNA)	81	69	94	**64**

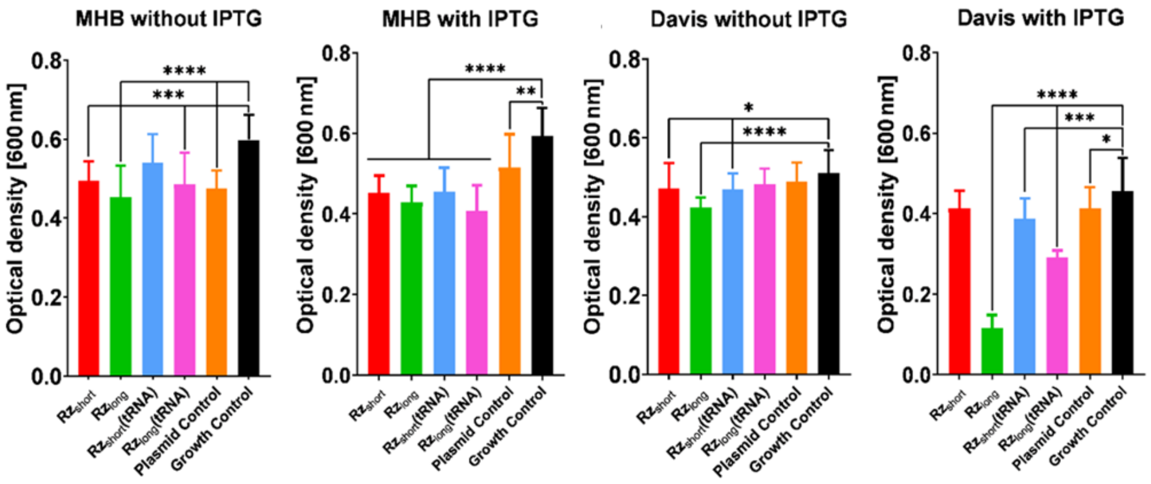

Supplementary Figure S15. Growth of *E. coli* BL21(DE3) containing plasmids with different ribozymes, measured by optical density in microplates after 24-hour cultivation in MHB and Davis media. Mean values ± SEM are shown (n = 6-18). Statistical comparisons are relative to the Growth Control: **** P < 0.0001, *** P < 0.001, ** P < 0.01, * P < 0.05; other differences are not significant.

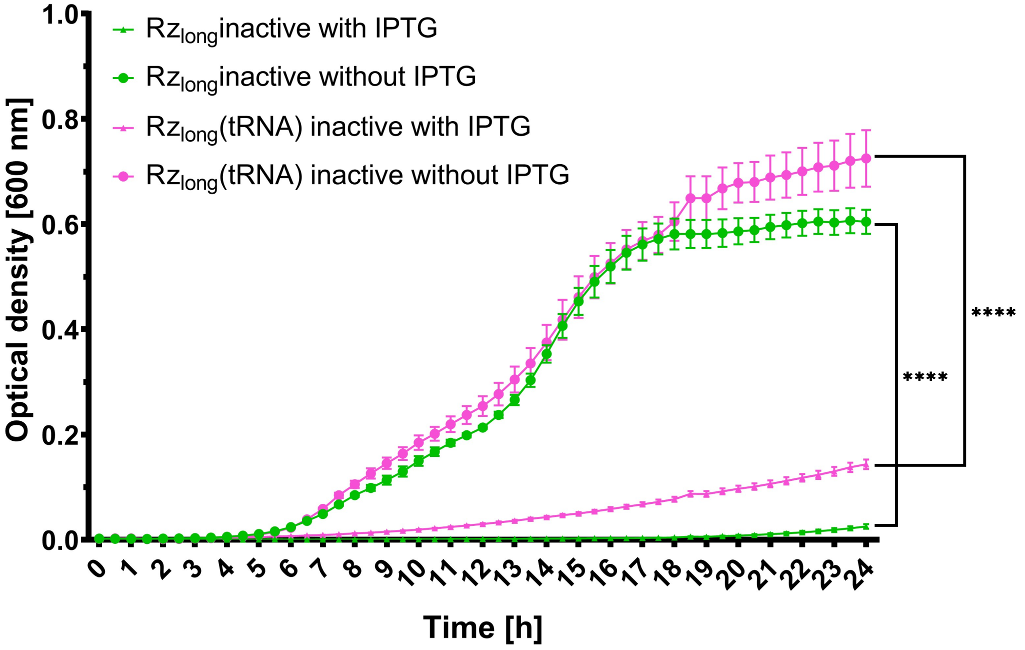

Supplementary Figure S16. Effect of IPTG induction on the 24-hour growth kinetics of *E. coli* BL21(DE3) grown in microplates in Davis medium. IPTG was added at the beginning of the experiments. Data are presented as the mean ± SEM (n = 3 for non-induced cultures; n = 21 for IPTG-induced cultures). For each ribozyme-containing construct, the difference in optical density between non-induced and IPTG-induced cultures is statistically significant (**** P < 0.0001).

Supplementary Table S4. Percentage of growth of *E. coli* BL21(DE3) cultures expressing different ribozymes relative to the Growth Control after 24 hours of cultivation. The Growth Control optical density was set as the 100 % reference. The suffixes 4A and 3A denote ribozymes containing four or three adenines in the catalytic core, respectively.

Conditions / Culture	Davis with IPTG [%] 4A	Davis with IPTG [%] 3A
Growth Control	100	100
Plasmid Control	103	103
Rz_{short}	94	92
Rz_{long}	**57**	**33**
Rz_{short}(tRNA)	97	98
Rz_{long}(tRNA)	**52**	**57**

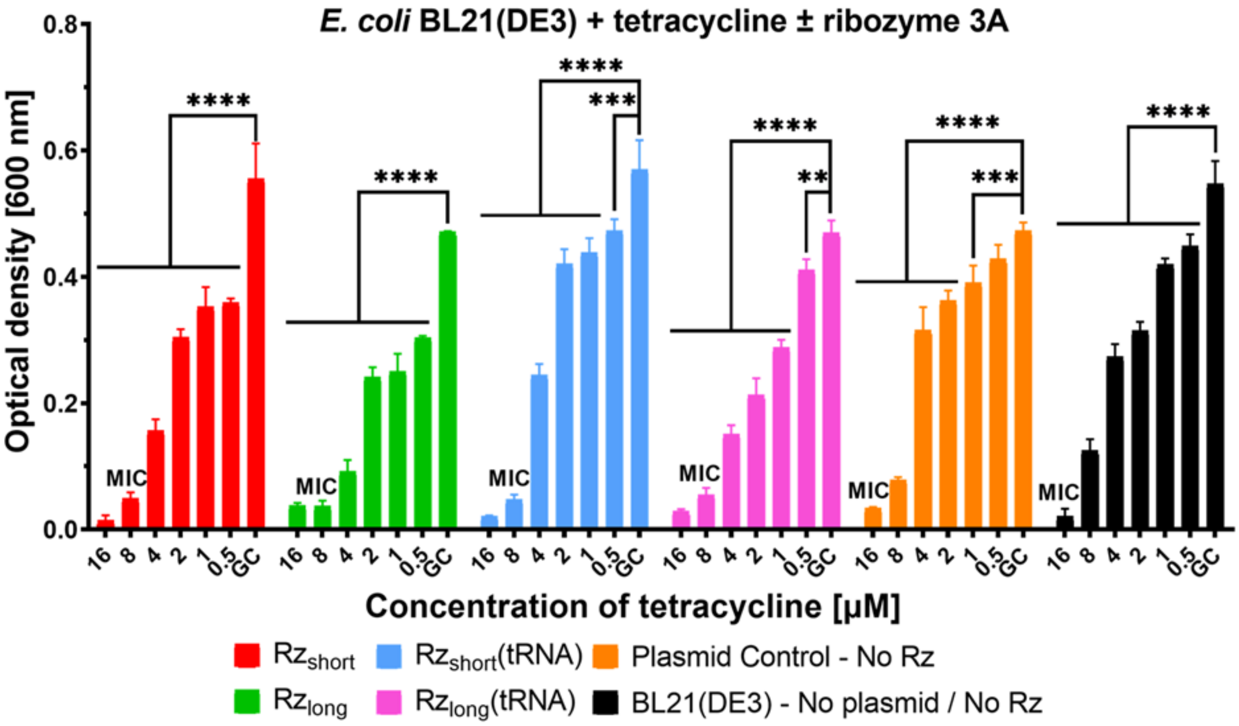

Supplementary Figure S17. Growth of *E. coli* cultures expressing the Rz 3A variants, in MHB medium, measured after a 20 h incubation with varying tetracycline concentrations. Data are presented as the mean ± SEM (n = 2-5). Statistical comparisons are relative to the Growth Control (GC): **** $P < 0.0001$, *** $P < 0.001$, and ** $P < 0.01$; other differences are not significant. MIC indicated the Minimum Inhibitory Concentration of tetracycline.

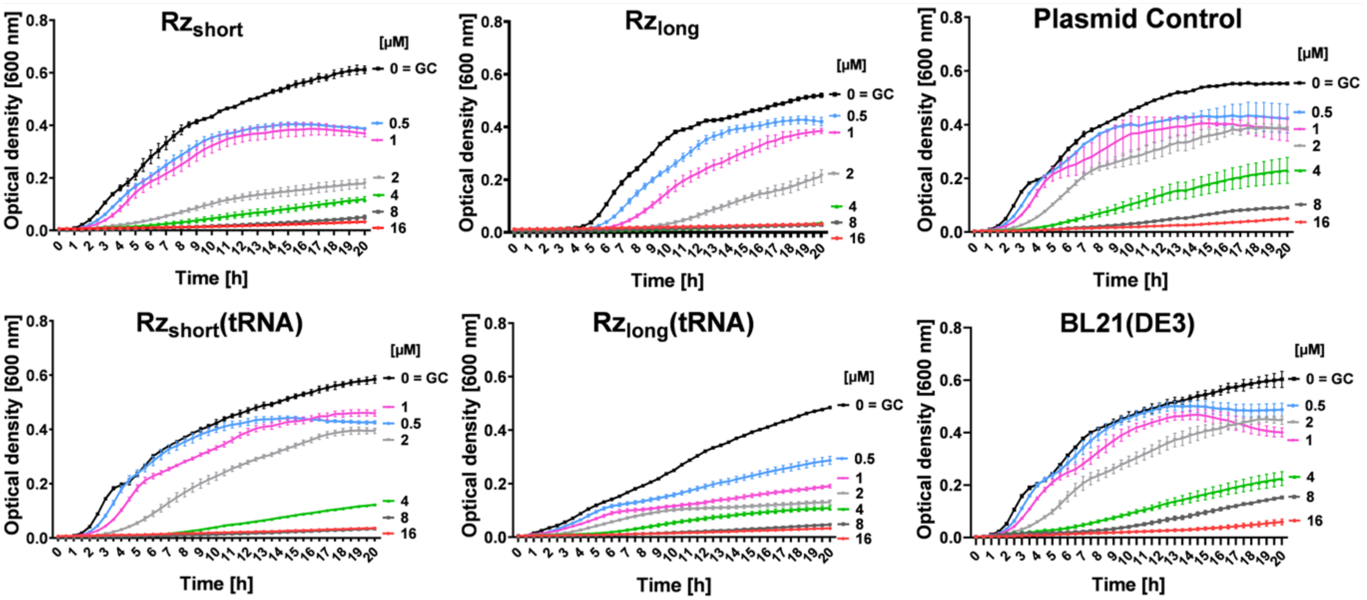

Supplementary Figure S18. Growth kinetics of *E. coli* BL21(DE3) cultures expressing Rz_4A variants. Bacteria were grown in microplates in MHB medium containing tetracycline and under IPTG induction, which was added at the beginning of the experiment. Mean values ± SEM are shown (n = 2-5).

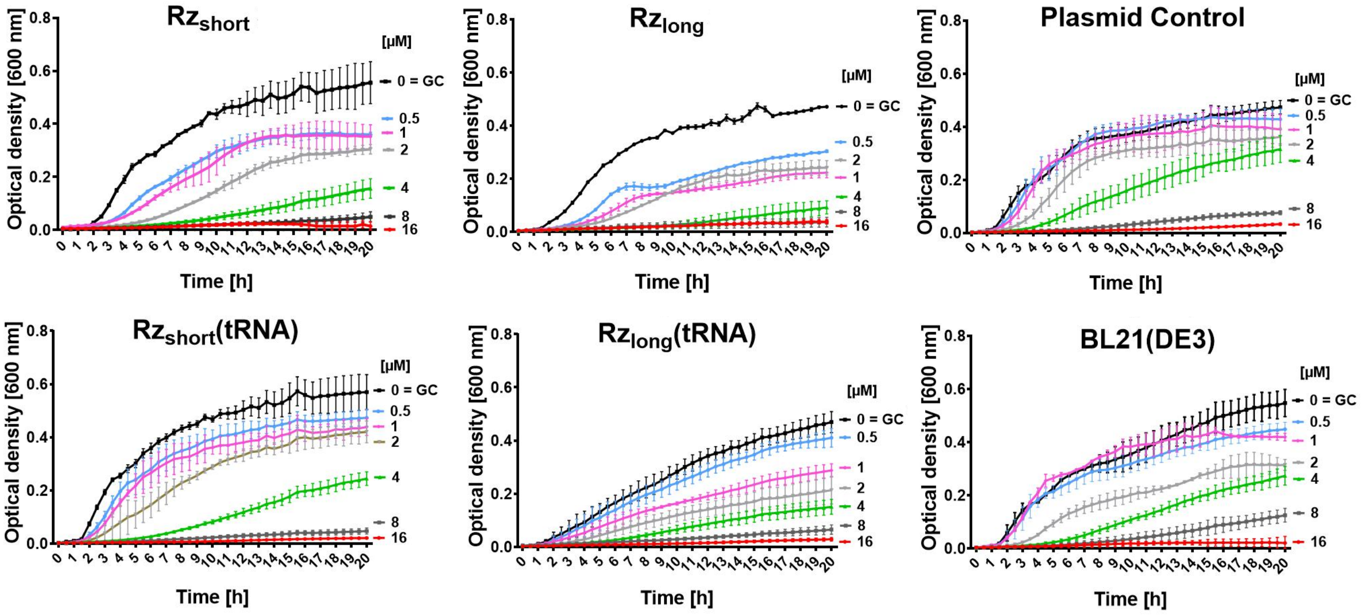

Supplementary Figure S19. Growth kinetics of *E. coli* BL21(DE3) cultures expressing Rz_3A variants. Bacteria were grown in microplates in MHB medium containing tetracycline and under IPTG induction, which was added at the beginning of the experiment. Mean values ± SEM are shown (n = 2-5).

Supplementary references

Altschul, S. 1997. 'Gapped BLAST and PSI-BLAST: A New Generation of Protein Database Search Programs'. *Nucleic Acids Research* 25(17):3389–3402. doi: 10.1093/nar/25.17.3389.

Chaudhuri, Roy R., Mohammed Sebaihia, Jon L. Hobman, Mark A. Webber, Denisse L. Leyton, Martin D. Goldberg, Adam F. Cunningham, Anthony Scott-Tucker, Paul R. Ferguson, Christopher M. Thomas, and et al. 2010. 'Complete Genome Sequence and Comparative Metabolic Profiling of the Prototypical Enteroaggregative Escherichia Coli Strain 042'. *PLoS ONE* 5(1):e8801. doi: 10.1371/journal.pone.0008801.

Chen, Swaine L., Chia-Seui Hung, Jian Xu, Christopher S. Reigstad, Vincent Magrini, Aniko Sabo, Darin Blasiar, Tamberlyn Bieri, Rekha R. Meyer, Philip Ozersky, and et al. 2006. 'Identification of Genes Subject to Positive Selection in Uropathogenic Strains of *Escherichia Coli* : A Comparative Genomics Approach'. *Proceedings of the National Academy of Sciences* 103(15):5977–82. doi: 10.1073/pnas.0600938103.

Cooper, Kerry K., Robert E. Mandrell, Jacqueline W. Louie, Jonas Korlach, Tyson A. Clark, Craig T. Parker, Steven Huynh, Patrick S. Chain, Sanaa Ahmed, and Michelle Qiu Carter. 2014. 'Comparative Genomics of Enterohemorrhagic Escherichia Coli O145:H28 Demonstrates a Common Evolutionary Lineage with Escherichia Coli O157:H7'. *BMC Genomics* 15(1):17. doi: 10.1186/1471-2164-15-17.

Crossman, Lisa C., Roy R. Chaudhuri, Scott A. Beatson, Timothy J. Wells, Mickael Desvaux, Adam F. Cunningham, Nicola K. Petty, Vivienne Mahon, Carl Brinkley, Jon L. Hobman, and et al. 2010. 'A Commensal Gone Bad: Complete Genome Sequence of the Prototypical Enterotoxigenic *Escherichia Coli* Strain H10407'. *Journal of Bacteriology* 192(21):5822–31. doi: 10.1128/JB.00710-10.

Forde, Brian M., Nouri L. Ben Zakour, Mitchell Stanton-Cook, Minh-Duy Phan, Makrina Totsika, Kate M. Peters, Kok Gan Chan, Mark A. Schembri, Mathew Upton, and Scott A. Beatson. 2014. 'The Complete Genome Sequence of Escherichia Coli EC958: A High Quality Reference Sequence for the Globally Disseminated Multidrug Resistant E. Coli O25b:H4-ST131 Clone'. *PLoS ONE* 9(8):e104400. doi: 10.1371/journal.pone.0104400.

Hall, T. A. 1999. 'BioEdit: A User-Friendly Biological Sequence Alignment Editor and Analysis Program for Windows 95/98/NT'. *Nucleic Acids Symposium Series* 41:95–98.

Iguchi, Atsushi, Nicholas R. Thomson, Yoshitoshi Ogura, David Saunders, Tadasuke Ooka, Ian R. Henderson, David Harris, M. Asadulghani, Ken Kurokawa, Paul Dean, and et al. 2009. 'Complete Genome Sequence and Comparative Genome Analysis of Enteropathogenic *Escherichia Coli* O127:H6 Strain E2348/69'. *Journal of Bacteriology* 191(1):347–54. doi: 10.1128/JB.01238-08.

Jeong, Haeyoung, Valérie Barbe, Choong Hoon Lee, David Vallenet, Dong Su Yu, Sang-Haeng Choi, Arnaud Couloux, Seung-Won Lee, Sung Ho Yoon, Laurence Cattolico, and et al. 2009. 'Genome Sequences of Escherichia Coli B Strains REL606 and BL21(DE3)'. *Journal of Molecular Biology* 394(4):644–52. doi: 10.1016/j.jmb.2009.09.052.

Kanehisa, M. 2000. 'KEGG: Kyoto Encyclopedia of Genes and Genomes'. *Nucleic Acids Research* 28(1):27–30. doi: 10.1093/nar/28.1.27.

Kyle, Jennifer L., Craig A. Cummings, Craig T. Parker, Beatriz Quiñones, Paolo Vatta, Elizabeth Newton, Steven Huynh, Michelle Swimley, Lovorka Degoricija, and Melissa Barker. 2012. ‘Escherichia Coli Serotype O55:H7 Diversity Supports Parallel Acquisition of Bacteriophage at Shiga Toxin Phage Insertion Sites during Evolution of the O157:H7 Lineage’. *Journal of Bacteriology* 194(8):1885–96. doi: 10.1128/JB.00120-12.

Makino, K. 1998. ‘Complete Nucleotide Sequences of 93-Kb and 3.3-Kb Plasmids of an Enterohemorrhagic Escherichia Coli O157:H7 Derived from Sakai Outbreak’. *DNA Research* 5(1):1–9. doi: 10.1093/dnares/5.1.1.

Perna, Nicole T., Guy Plunkett, Valerie Burland, Bob Mau, Jeremy D. Glasner, Debra J. Rose, George F. Mayhew, Peter S. Evans, Jason Gregor, Heather A. Kirkpatrick, and et al. 2001. ‘Genome Sequence of Enterohaemorrhagic Escherichia Coli O157:H7’. *Nature* 409(6819):529–33. doi: 10.1038/35054089.

Rasko, David A., M. J. Rosovitz, Garry S. A. Myers, Emmanuel F. Mongodin, W. Florian Fricke, Pawel Gajer, Jonathan Crabtree, Mohammed Sebaihia, Nicholas R. Thomson, Roy Chaudhuri, and et al. 2008. ‘The Pangenome Structure of *Escherichia Coli* : Comparative Genomic Analysis of *E. Coli* Commensal and Pathogenic Isolates’. *Journal of Bacteriology* 190(20):6881–93. doi: 10.1128/JB.00619-08.

Shepard, Sara M., Jessica L. Danzeisen, Richard E. Isaacson, Torsten Seemann, Mark Achtman, and Timothy J. Johnson. 2012. ‘Genome Sequences and Phylogenetic Analysis of K88- and F18-Positive Porcine Enterotoxigenic Escherichia Coli’. *Journal of Bacteriology* 194(2):395–405. doi: 10.1128/JB.06225-11.

Welch, R. A., V. Burland, G. Plunkett, P. Redford, P. Roesch, D. Rasko, E. L. Buckles, S. R. Liou, A. Boutin, J. Hackett, and et al. 2002. ‘Extensive Mosaic Structure Revealed by the Complete Genome Sequence of Uropathogenic *Escherichia Coli*’. *Proceedings of the National Academy of Sciences* 99(26):17020–24. doi: 10.1073/pnas.252529799.

Zhou, Kang, Lihan Zhou, Qing ’En Lim, Ruiyang Zou, Gregory Stephanopoulos, and Heng-Phon Too. 2011. ‘Novel Reference Genes for Quantifying Transcriptional Responses of Escherichia Coli to Protein Overexpression by Quantitative PCR’. *BMC Molecular Biology* 12(1):18. doi: 10.1186/1471-2199-12-18.
